# Post-stenting fractional flow reserve vs coronary angiography for optimization of percutaneous coronary intervention (TARGET-FFR)

**DOI:** 10.1093/eurheartj/ehab449

**Published:** 2021-07-19

**Authors:** Damien Collison, Matthaios Didagelos, Muhammad Aetesam-ur-Rahman, Samuel Copt, Robert McDade, Peter McCartney, Thomas J Ford, John McClure, Mitchell Lindsay, Aadil Shaukat, Paul Rocchiccioli, Richard Brogan, Stuart Watkins, Margaret McEntegart, Richard Good, Keith Robertson, Patrick O’Boyle, Andrew Davie, Adnan Khan, Stuart Hood, Hany Eteiba, Colin Berry, Keith G Oldroyd

**Affiliations:** West of Scotland Regional Heart & Lung Centre, Golden Jubilee National Hospital, Agamemnon Street, Clydebank, G81 4DY, UK; Institute of Cardiovascular & Medical Sciences, University of Glasgow, 126 University Place, Glasgow, G12 8TA, UK; West of Scotland Regional Heart & Lung Centre, Golden Jubilee National Hospital, Agamemnon Street, Clydebank, G81 4DY, UK; West of Scotland Regional Heart & Lung Centre, Golden Jubilee National Hospital, Agamemnon Street, Clydebank, G81 4DY, UK; University of Geneva, 24 rue de Général-Dufour, 1211 Genève 4, Switzerland; West of Scotland Regional Heart & Lung Centre, Golden Jubilee National Hospital, Agamemnon Street, Clydebank, G81 4DY, UK; West of Scotland Regional Heart & Lung Centre, Golden Jubilee National Hospital, Agamemnon Street, Clydebank, G81 4DY, UK; Institute of Cardiovascular & Medical Sciences, University of Glasgow, 126 University Place, Glasgow, G12 8TA, UK; Institute of Cardiovascular & Medical Sciences, University of Glasgow, 126 University Place, Glasgow, G12 8TA, UK; Institute of Cardiovascular & Medical Sciences, University of Glasgow, 126 University Place, Glasgow, G12 8TA, UK; West of Scotland Regional Heart & Lung Centre, Golden Jubilee National Hospital, Agamemnon Street, Clydebank, G81 4DY, UK; West of Scotland Regional Heart & Lung Centre, Golden Jubilee National Hospital, Agamemnon Street, Clydebank, G81 4DY, UK; West of Scotland Regional Heart & Lung Centre, Golden Jubilee National Hospital, Agamemnon Street, Clydebank, G81 4DY, UK; West of Scotland Regional Heart & Lung Centre, Golden Jubilee National Hospital, Agamemnon Street, Clydebank, G81 4DY, UK; West of Scotland Regional Heart & Lung Centre, Golden Jubilee National Hospital, Agamemnon Street, Clydebank, G81 4DY, UK; Institute of Cardiovascular & Medical Sciences, University of Glasgow, 126 University Place, Glasgow, G12 8TA, UK; West of Scotland Regional Heart & Lung Centre, Golden Jubilee National Hospital, Agamemnon Street, Clydebank, G81 4DY, UK; Institute of Cardiovascular & Medical Sciences, University of Glasgow, 126 University Place, Glasgow, G12 8TA, UK; West of Scotland Regional Heart & Lung Centre, Golden Jubilee National Hospital, Agamemnon Street, Clydebank, G81 4DY, UK; Institute of Cardiovascular & Medical Sciences, University of Glasgow, 126 University Place, Glasgow, G12 8TA, UK; West of Scotland Regional Heart & Lung Centre, Golden Jubilee National Hospital, Agamemnon Street, Clydebank, G81 4DY, UK; West of Scotland Regional Heart & Lung Centre, Golden Jubilee National Hospital, Agamemnon Street, Clydebank, G81 4DY, UK; West of Scotland Regional Heart & Lung Centre, Golden Jubilee National Hospital, Agamemnon Street, Clydebank, G81 4DY, UK; West of Scotland Regional Heart & Lung Centre, Golden Jubilee National Hospital, Agamemnon Street, Clydebank, G81 4DY, UK; West of Scotland Regional Heart & Lung Centre, Golden Jubilee National Hospital, Agamemnon Street, Clydebank, G81 4DY, UK; West of Scotland Regional Heart & Lung Centre, Golden Jubilee National Hospital, Agamemnon Street, Clydebank, G81 4DY, UK; Institute of Cardiovascular & Medical Sciences, University of Glasgow, 126 University Place, Glasgow, G12 8TA, UK; West of Scotland Regional Heart & Lung Centre, Golden Jubilee National Hospital, Agamemnon Street, Clydebank, G81 4DY, UK; Institute of Cardiovascular & Medical Sciences, University of Glasgow, 126 University Place, Glasgow, G12 8TA, UK; West of Scotland Regional Heart & Lung Centre, Golden Jubilee National Hospital, Agamemnon Street, Clydebank, G81 4DY, UK; Institute of Cardiovascular & Medical Sciences, University of Glasgow, 126 University Place, Glasgow, G12 8TA, UK

**Keywords:** Ischaemic heart disease, Coronary physiology, Fractional flow reserve, Functional optimization, PCI Optimization

## Abstract

**Aims:**

A fractional flow reserve (FFR) value ≥0.90 after percutaneous coronary intervention (PCI) is associated with a reduced risk of adverse cardiovascular events. TARGET-FFR is an investigator-initiated, single-centre, randomized controlled trial to determine the feasibility and efficacy of a post-PCI FFR-guided optimization strategy vs. standard coronary angiography in achieving final post-PCI FFR values ≥0.90.

**Methods and results:**

After angiographically guided PCI, patients were randomized 1:1 to receive a physiology-guided incremental optimization strategy (PIOS) or a blinded coronary physiology assessment (control group). The primary outcome was the proportion of patients with a final post-PCI FFR ≥0.90. Final FFR ≤0.80 was a prioritized secondary outcome. A total of 260 patients were randomized (131 to PIOS, 129 to control) and 68.1% of patients had an initial post-PCI FFR <0.90. In the PIOS group, 30.5% underwent further intervention (stent post-dilation and/or additional stenting). There was no significant difference in the primary endpoint of the proportion of patients with final post-PCI FFR ≥0.90 between groups (PIOS minus control 10%, 95% confidence interval −1.84 to 21.91, *P* = 0.099). The proportion of patients with a final FFR ≤0.80 was significantly reduced when compared with the angiography-guided control group (−11.2%, 95% confidence interval −21.87 to −0.35], *P* = 0.045).

**Conclusion:**

Over two-thirds of patients had a physiologically suboptimal result after angiography-guided PCI. An FFR-guided optimization strategy did not significantly increase the proportion of patients with a final FFR ≥0.90, but did reduce the proportion of patients with a final FFR ≤0.80.

## Graphical Abstract

**Figure:**
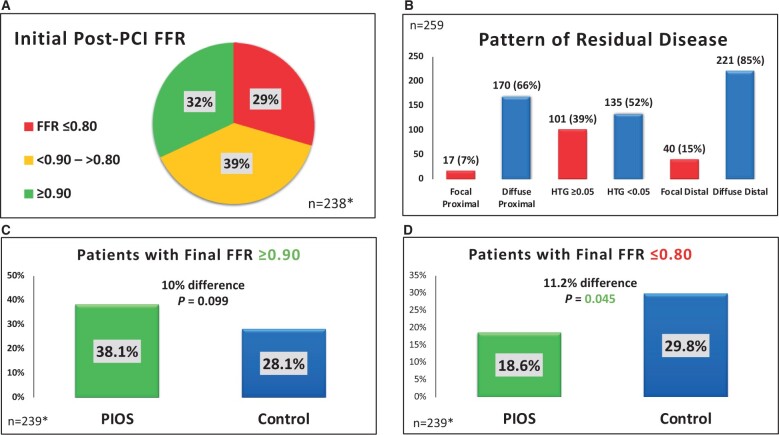
Findings of initial post-percutaneous coronary intervention fractional flow reserve and pullback assessments. (*A*) Post-percutaneous coronary intervention fractional flow reserve results following standard-of-care stenting [*238/260 (92%) with core lab-adjudicated post-percutaneous coronary intervention fractional flow reserve results available for analysis]. (*B*) Summary findings of 259 initial post-percutaneous coronary intervention fractional flow reserve pullback assessments (pre-randomization) demonstrating the patterns of residual disease in the study vessels. Protocol-defined targets for additional optimization measure in red bars. Multiple findings may have co-existed within individual vessels. Focal lesion defined as an abrupt pressure drop ≥0.05 fractional flow reserve units on pullback. (*C*) Primary endpoint—Proportion of patients with final post-percutaneous coronary intervention fractional flow reserve value ≥0.90. (*D*) Secondary endpoint—Proportion of patients with final post-percutaneous coronary intervention fractional flow reserve ≤0.80. Diffuse Distal, diffuse pressure gradient distal to stented segment; Diffuse Proximal, diffuse pressure gradient proximal to stented segment; Focal Distal, focal pressure drop distal to stented segment; Focal Proximal, Focal pressure drop proximal to stented segment; FFR, fractional flow reserve; HTG, hyperaemic trans-stent gradient; PCI, percutaneous coronary intervention.

**See page 4669 for the editorial comment for this article ‘We need intracoronary physiology guidance before percutaneous coronary intervention, but do we need it post-stenting?’, by D. Erlinge and M. Götberg, https://doi.org/10.1093/eurheartj/ehab525.**

## Introduction

Fractional flow reserve (FFR)-guided percutaneous coronary intervention (PCI), as compared with angiography-guided PCI, reduces unnecessary stenting and lowers the risk of myocardial infarction at 2 years.^1^ The utility of measuring FFR after PCI is less certain but post-PCI FFR values are reported to be inversely associated with adverse cardiac events.^2^ In a meta-analysis of 7470 patients, post-PCI FFR values ≥0.90 were associated with a lower risk of repeat PCI and major adverse cardiovascular events (MACE).^3^ However, post-PCI FFR is rarely assessed in clinical practice. Potential barriers may be the assumptions that (i) the incidence of suboptimal FFR results post-PCI is low; (ii) where this occurs, it is primarily related to residual diffuse disease in the vessel and, accordingly, (iii) there is limited scope to improve the FFR result through further intervention.

In fact, data on post-PCI FFR vary widely and the true incidence of suboptimal FFR after stenting is unknown. The reported proportion of patients achieving an optimal post-PCI FFR of ≥0.90 ranges from 21% to 100%.^4–14^ Furthermore, from previous reports, a post-PCI FFR that remains below the clinical threshold for revascularization (FFR ≤0.80) despite angiographically successful PCI may occur in <1% or as many 36.5% of patients.^11–24^ Registry data indicate that identifying and intervening on coronary arteries with suboptimal post-PCI FFR can achieve improvements in the final FFR value but with varying degrees of success.^14^ ^,^ ^25^ ^,^ ^26^ The Trial of Angiography vs. pressure-Ratio-Guided Enhancement Techniques—Fractional Flow Reserve (TARGET-FFR) was designed to assess the efficacy of a post-PCI physiology-guided incremental optimization strategy (PIOS) vs. standard angiographic guidance in achieving final post-PCI FFR values ≥0.90.

## Methods

### Study design

TARGET-FFR was a prospective, single-centre, randomized, controlled, parallel group, blinded, clinical trial conducted at the Golden Jubilee National Hospital in Glasgow, UK. The study complies with the Declaration of Helsinki and the West of Scotland Research Ethics Committee 3 gave it a favourable opinion on 18 August 2017 (reference 17/WS/0153). The trial was sponsored and monitored by the NHS National Waiting Times Centre. Coronary physiology data were adjudicated and validated by a core laboratory (CoreAalst BV, Aalst, Belgium) blinded to treatment group assignment. Clinical endpoints were adjudicated by an independent Clinical Events Committee. The study rationale and design have been described previously.^27^ The study is registered on ClinicalTrials.gov (Identifier: NCT03259815).

### Participants

Patients eligible for the trial were >18 years of age and undergoing PCI for either stable angina, medically stabilized non-ST-elevation myocardial infarction (NSTEMI), or staged completion of non-culprit vessel revascularization following either NSTEMI or ST-elevation myocardial infarction (STEMI). Since the primary objective was focused on PCI optimization, the target population was unrestricted and patients with either stable or acute coronary syndromes (ACS) were included. Exclusion criteria were PCI to a coronary bypass graft, PCI of an in-stent restenosis lesion, PCI to a target artery providing Rentrop grade 2 or 3 collateral blood supply to another vessel, inability to receive adenosine (e.g. severe reactive airway disease, marked hypotension, or advanced atrioventricular block without pacemaker), recent (within 1 week prior to cardiac catheterization) STEMI in any arterial distribution (not specifically target lesion), severe cardiomyopathy (left ventricular ejection fraction <30%), and renal insufficiency such that an additional 20–30 mL of contrast would, in the opinion of the operator, pose unwarranted risk to the patient. Patients were invited to participate prior to undergoing coronary angiography and were enrolled only after providing written informed consent. All subjects completed Seattle Angina Questionnaires (SAQ-7) and the EQ-5D-5L health-related quality of life questionnaire prior to their procedure.

### Randomization and masking

Patients proceeding to PCI had invasive coronary physiology assessment prior to intervention. Once the operator declared the PCI procedure to be complete, patients meeting all inclusion and exclusion criteria were eligible for randomization. This was performed in the catheterization laboratory using a 1:1 variable block (2, 4, 6) randomization method generated through a secure (ISO 27001 and 9001 compliant) web-based platform. The timing of the randomization before FFR measurement was intended to limit bias and prevent selection of patients for randomization when FFR was already known. An operator-blinded invasive coronary physiology assessment was then performed. The coronary physiology data were obscured and the digital interface was visible only to the researcher (D.C.) who advised on measurement quality. This concluded the procedure for all patients in the control group and those in the PIOS intervention group with FFR ≥0.90. In patients randomized to the PIOS group with post-PCI FFR <0.90, operators reviewed the measurements and planned additional intervention based on the findings of the FFR pullback assessment. Following these additional optimization measures, physiology assessment was repeated and the procedure was completed. Final coronary physiology results were not disclosed to patients.

### Procedures

The PCI procedure was undertaken according to operator judgement (including the use of intracoronary imaging) in line with contemporary standards of care. All PCI procedures were performed using drug-eluting stents.

Coronary physiology measurements were acquired using the PressureWire X Guidewire (Abbott Laboratories, IL, USA) and analysed in real time (CoroFlow v3.0, Coroventis Research AB, Uppsala, Sweden). Following administration of a 200 μg bolus of intracoronary nitrate to the study artery, the pressure wire sensor was positioned at the tip of the guide catheter and equalized with the aortic pressure. The pressure wire was then advanced to position the sensor in the distal third of the vessel. Hyperaemia was induced by infusion of adenosine into an antecubital vein at a rate of 140 μg/kg/min. In addition to resting Pd/Pa, the following non-hyperaemic pressure ratios were measured: diastolic pressure ratio (dPR)—the Pd/Pa ratio of the averaged Pa and Pd values measured during the entire diastolic period of five consecutive cardiac cycles; resting full-cycle ratio (RFR)—the lowest Pd/Pa ratio over an entire cardiac cycle averaged over five consecutive cycles. Microvascular function was simultaneously assessed with FFR. Using a thermodilution technique, coronary flow reserve (CFR—the ratio of resting to hyperaemic coronary flow) and the index of microcirculatory resistance (IMR—the product of mean hyperaemic distal coronary pressure and mean hyperaemic transit time) were calculated as previously described.^27^ Fractional flow reserve was measured during stable hyperaemia with the sensor positioned as far distally in the vessel as practical. Finally, a hyperaemic pressure wire pullback assessment was performed and the sensor returned to the tip of the guide catheter to assess for pressure drift. If there was drift of >0.03 units, repeat measurements were requested. Using the CoroFlow software, the research cardiologist annotated the hyperaemic pullback recording to co-register the anatomical landmarks during fluoroscopy-guided pullback of the pressure wire (distal and proximal stent edges, the position of relevant side branches and the tip of the guiding catheter, etc.). This allowed calculation of the hyperaemic trans-stent gradient (HTG) and localization of residual pressure gradients proximal or distal to the stented segment.

**Figure 1:**
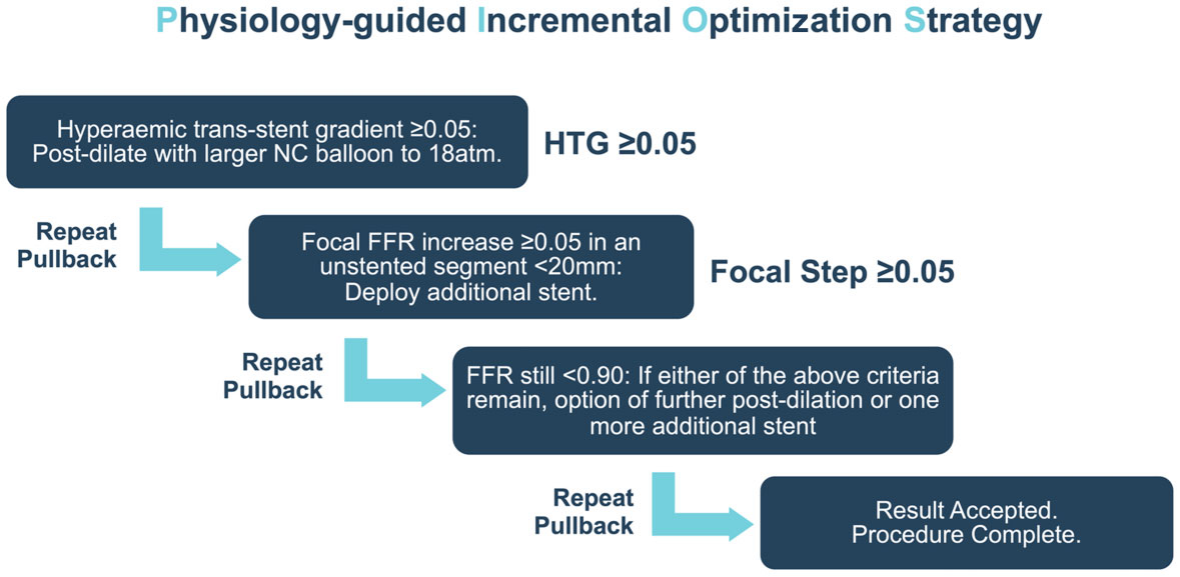
Physiology-guided incremental optimization strategy. FFR, fractional flow reserve; HTG, hyperaemic trans-stent gradient; NC, non-compliant.

Patients in the PIOS group with post-PCI FFR <0.90 had their coronary physiology findings disclosed to the operator. Based on the clinical interpretation of the FFR changes (pressure loss) in the treated artery post-PCI, the operator then followed the PIOS algorithm to optimize the final PCI result (*Figure 1*). If the residual pressure gradient reflected diffuse atherosclerosis with no focal step-changes in pressure gradient, the result was accepted and no optimization attempted.

Three months after PCI, participants were invited to complete questionnaires for anginal symptoms (SAQ-7) and health status (EQ-5D-5L). The questionnaires were administered by telephone or mail by a research nurse blinded to the randomized group allocation and the physiology results. Clinical outcomes at 1-year post-PCI were assessed by electronic health record linkage.

For the purposes of the primary and relevant secondary endpoints, coronary physiology data underwent *post* *hoc* adjudication by an independent core laboratory (CoreAalst BV, Aalst, Belgium). Each individual tracing was assessed for quality based on pre-specified criteria and received a binary decision regarding adequate quality for inclusion in the final analysis. Fractional flow reserve, CFR and IMR were calculated independently from the corresponding recordings. Further details on the core lab adjudication process are available in the Supplementary material online.

### Outcomes

The primary endpoint of the study was the proportion of patients with a final post-PCI FFR result ≥0.90. Secondary endpoints were proportion of patients with final FFR ≤0.80 (the guideline-directed threshold for revascularization^28^); change from baseline SAQ-7 scores at 3 months; change from baseline EQ-5D-5L scores at 3 months; rate of target vessel failure and its components (cardiac death, myocardial infarction, stent thrombosis, unplanned re-hospitalization with target vessel revascularization) at 1 year; proportion with final post-PCI dPR ≥0.90; proportion with final post-PCI RFR ≥0.90; proportion with final post-PCI CFR ≥2.0; proportion with final post-PCI IMR >25; proportion with final post-PCI IMRc >25; absolute and relative change in FFR (pre-to-final); absolute and relative change in dPR (pre-to-final); absolute and relative change in RFR (pre-to-final); absolute and relative change in CFR (pre-to-final); absolute and relative change in resting transit time (pre-to-final); absolute and relative change in hyperaemic transit time (pre-to-final); absolute and relative change in IMR (pre-to-final); absolute and relative change in IMRc (pre-to-final).

Safety analyses included: procedure duration, fluoroscopy dose; contrast material dose; adenosine dose; incidence of the following procedural complications—coronary artery dissection, side branch occlusion, no flow/slow flow, haematoma >5 cm, and Type 4a myocardial infarction.

### Statistical analysis

There are no prior randomized clinical trials of post-PCI FFR optimization strategies. In a registry of 664 vessels from 574 patients, 20.6% (137/664) underwent additional intervention based on post-PCI FFR ≤0.80 and/or operator discretion with 87/137 (63.5%) having post-PCI FFR ≤0.80. Optimization increased the final overall proportion of patients with FFR >0.91 by 9%.^25^ Since a post-PCI FFR threshold of 0.90 is associated with prognosis, we hypothesized that a systematic approach to measure FFR post-PCI to detect the subgroup of patients with an FFR <0.90 and then intervene with a PIOS procedure, would increase the proportion of patients with a final FFR ≥0.90 by 20%. We believed a change of this magnitude would be clinically relevant. On this basis, a sample size of 130 patients per group was required to have 90% power to detect a 20% difference between groups at the 5% significance level. Patients with stable angina or NSTEMI attending our institution for diagnostic coronary angiography proceed to PCI during the same procedure in approximately 40% of cases. Therefore, we estimated that approximately 650 patients would need to be enrolled in the study in order to randomize 260 following standard-of-care PCI.

Continuous variables are presented as mean ± SD, and categorical data as counts and percentages. A two-sample *t*-test was used to compare patient-level characteristics with continuous variables. Categorical variables were compared using a χ^2^ test without continuity correction. Whenever appropriate, a Fisher’s exact test was used instead. 95% confidence intervals (CI) for between-group differences were calculated using the Wald method without continuity correction. Comparison of pre- and post-PCI values were performed using an ANCOVA model on the parameter’s percent change adjusted for the treatment group and baseline value.

## Results

**Figure 2:**
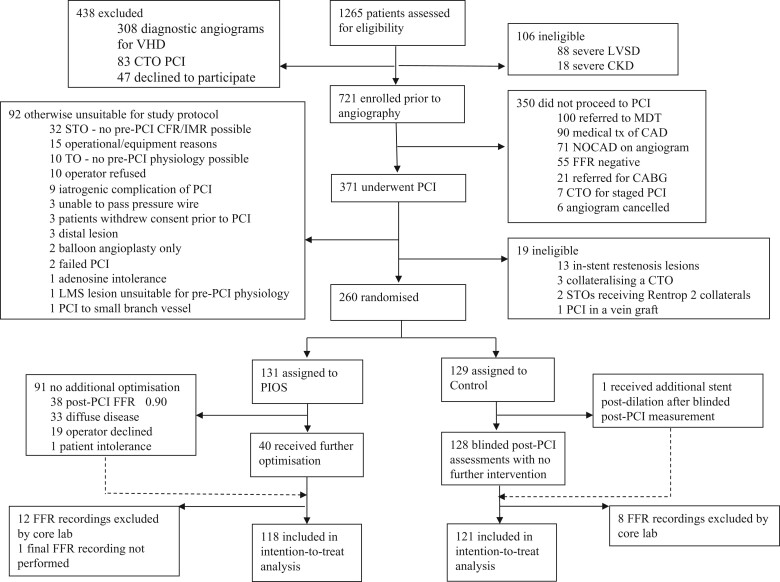
Trial profile. CABG, coronary artery bypass graft surgery; CAD, coronary artery disease; CFR, coronary flow reserve; CKD, chronic kidney disease; CTO, chronic total occlusion; FFR, fractional flow reserve; IMR, index of microcirculatory resistance; LMS, left main stem; LVSD, left ventricular systolic dysfunction; MDT, multi-disciplinary team meeting; NOCAD, no obstructive coronary artery disease; PCI, percutaneous coronary intervention; PIOS, physiology-guided incremental optimization strategy; STO, sub-total occlusion; TO, total occlusion; VHD, valvular heart disease.

Between 22 February 2018 and 22 November 2019, 1265 patients attending for coronary angiography and/or PCI were assessed for eligibility (*Figure 2*). Of these, 721 were enrolled in the trial before their procedures. Following PCI, 260 patients were randomized to either the PIOS intervention group or control group (blinded physiology assessment). Clinical and procedural characteristics at baseline were evenly distributed between the randomized groups (*Tables 1 and 2*).

**Table 1:** Baseline characteristics

	Total (*n* = 260)	PIOS (*n* = 131)	Control (*n* = 129)	*P*-value
Male sex	226 (86.9)	117 (89.3)	109 (84.5)	0.25
Age (years)	59 ± 12	58 ± 12	60 ± 13	0.17
BMI (kg/m^2^)	29.1 ± 5.7	28.9 ± 6	29.4 ± 5.3	0.34
Hypertension	116 (44.6)	58 (44.3)	58 (45)	0.91
Hypercholesterolaemia	146 (56.2)	72 (55)	74 (57.4)	0.70
Diabetes	49 (18.8)	24 (18.3)	25 (19.4)	0.83
OHAs	42 (85.7)	21 (87.5)	21 (84)	1.00
Insulin	5 (10.2)	3 (12.5)	2 (8)	0.67
Atrial fibrillation	19 (7.3)	10 (7.6)	9 (7)	0.84
OAC	13 (68.4)	6 (60)	7 (77.8)	0.63
Previous TIA/stroke	17 (6.5)	8 (6.1)	9 (7)	0.78
CKD^a^	5 (1.9)	3 (2.3)	2 (1.6)	1.00
Family history of CAD	172 (66.2)	88 (67.2)	84 (65.1)	0.73
History of smoking	183 (70.4)	92 (70.2)	91 (70.5)	0.96
Type of smoker				0.44
Current	50 (27.3)	28 (30.4)	22 (24.2)	
Within past year	41 (22.4)	22 (23.9)	19 (20.9)	
Ex-smoker >1year	92 (50.3)	42 (45.7)	50 (54.9)	
Thyroid dysfunction	20 (7.7)	9 (6.9)	11 (8.5)	0.62
Heart failure	63 (24.2)	35 (26.7)	28 (21.7)	0.35
HFrEF	62 (98.4)	35 (100)	27 (96.4)	0.44
HFrEF Severity				0.21
Mild	43 (69.4)	22 (62.9)	21 (77.8)	
Moderate	19 (30.6)	13 (37.1)	6 (22.2)	
NYHA Class				0.42
I	44 (69.8)	23 (65.7)	21 (75)	
II	19 (30.2)	12 (34.3)	7 (25)	
Previous MI	95 (36.5)	50 (38.2)	45 (34.9)	0.58
Previous PCI	100 (38.5)	54 (41.2)	46 (35.7)	0.36
Previous CABG	1 (0.4)	1 (0.8)	0	1.00
Valvular heart disease^b^	8 (3.1)	2 (1.5)	6 (4.7)	0.17
Aortic stenosis	6 (2.3)	1 (0.8)	5 (3.9)	0.12
Mitral regurgitation	2 (0.8)	1 (0.8)	1 (0.8)	1.00
Angina	215 (82.7)	107 (81.7)	108 (83.7)	0.66
CCS Class				0.98
I	58 (27)	28 (26.2)	30 (27.8)	
II	101 (47)	51 (47.7)	50 (46.3)	
III	55 (25.6)	27 (25.2)	28 (25.9)	
IV	1 (0.5)	1 (0.9)	0	
Cardiac medications				
Single APT	253 (97.3)	128 (97.7)	125 (96.9)	0.72
Dual APT	185 (71.2)	97 (74.1)	88 (68.2)	0.30
OAC	16 (6.2)	8 (6.1)	8 (6.2)	0.98
Statin	250 (96.2)	127 (96.9)	123 (95.3)	0.54
Beta-blocker	237 (91.2)	121 (92.4)	116 (89.9)	0.49
CCB	52 (20)	22 (16.8)	30 (23.3)	0.19
ACEI	175 (67.3)	91 (69.5)	84 (65.1)	0.46
ARB	23 (8.9)	11 (8.4)	12 (9.3)	0.80
Diuretic	30 (11.5)	13 (9.9)	17 (13.2)	0.41
GTN spray use	123 (47.3)	61 (46.6)	62 (48.1)	0.81
Frequency of GTN use				0.73
Daily	30 (24.4)	13 (21.3)	17 (27.4)	
Weekly	67 (54.55)	34 (55.7)	32 (51.6)	
Monthly	27 (22)	14 (23)	13 (21)	
Oral Nitrate	69 (26.5)	26 (19.8)	43 (33.3)	0.01
Nicorandil	22 (8.5)	14 (10.7)	8 (6.2)	0.19
Ivabradine	5 (1.9)	3 (2.3)	2 (1.6)	1.00
No. of anti-anginal meds				0.65
0	9 (3.5)	4 (3.1)	5 (3.9)	
1	99 (38.1)	55 (42)	44 (34.1)	
2	114 (43.8)	55 (42)	59 (45.7)	
3	31 (11.9)	13 (9.9)	18 (14)	
4	7 (2.7)	4 (3.1)	3 (2.3)	

Values are *n* (%), or mean ± SD.

a All 5 patients had Stage 3a CKD (eGFR 45–59): Mild-moderate renal impairment.

b Degree of valve disease was either mild or moderate.

ACEI, angiotensin converting enzyme inhibitor; APT, antiplatelet therapy; ARB, angiotensin ii-receptor blocker; BMI, body mass index; CABG, coronary artery bypass grafting; CAD, coronary artery disease; CCB, calcium channel blocker; CCS, Canadian Cardiovascular Society; CKD, chronic kidney disease; eGFR, estimated glomerular filtration rate; GTN, glyceryl trinitrate; HFrEF, heart failure with reduced ejection fraction; MI, myocardial infarction; OAC, oral anticoagulant; OHAs, oral hypoglycaemic agents; PCI, percutaneous coronary intervention; PIOS, physiology-guided incremental optimization strategy.

**Table 2:** Procedural characteristics

	Total (*n* = 260)	PIOS (*n* = 131)	Control (*n* = 129)	*P*-value
Indication				
Stable angina	72 (27.7)	32 (24.4)	40 (31)	0.24
NSTE-ACS	101 (38.8)	50 (38.2)	51 (39.5)	0.82
Days post-MI	21 (17)	20 (19)	23 (15)	0.06
ACS-unstable angina	3 (1.2)	2 (1.5)	1 (0.8)	1.00
Staged PCI/completion of revascularization	84 (32.3)	47 (35.9)	37 (28.7)	0.22
Stable angina	16 (19)	8 (17)	8 (21.6)	0.98
Post-NSTEMI	22 (26.2)	10 (21.3)	12 (32.4)	0.63
Days since MI	67 (44)	64 (33)	80 (58)	0.67
Post-STEMI	46 (54.8)	29 (61.7)	17 (45.9)	0.06
Days since MI	69 ± 29	70 ± 31	66 ± 28	0.64
Multivessel PCI (%)	28 (10.8)	17 (13)	11 (8.5)	0.25
Target vessel				
LAD	149 (57.3)	75 (57.3)	74 (57.4)	0.98
RCA	67 (25.8)	28 (21.4)	39 (30.2)	0.10
LCx	33 (12.7)	20 (15.3)	13 (10.1)	0.21
OM	10 (3.8)	8 (6.1)	2 (1.6)	0.10
Diagonal	1 (0.4)	0	1 (0.8)	0.50
QCA diameter stenosis (%)	65.7 ± 15.1	65.85 ± 14.78	65.60 ± 15.51	0.89
QCA area stenosis (%)	85.8 ± 12.4	85.73 ± 12.86	85.80 ± 11.92	0.96
QCA lesion length (mm)	12.2 ± 5.9	11.96 ± 5.50	12.36 ± 6.37	0.59
Clinically instigated pressure wire	91 (35)	43 (32.8)	48 (37.2)	0.46
PCI performed on pressure wire	64 (24.6)	32 (24.4)	32 (24.8)	0.94
Pre-dilation of lesion	260 (100)	131 (100)	129 (100)	NS
Rotational atherectomy	7 (2.7)	2 (1.5)	5 (3.9)	0.24
Intravascular imaging	42 (16.2)	17 (13)	25 (19.4)	0.16
Imaging type				0.07
IVUS	34 (81)	16 (94.1)	18 (72)	
OCT	8 (19)	1 (5.9)	7 (28)	
Target lesion stent diameter (mm)	3.23 ± 0.43	3.21 ± 0.43	3.25 ± 0.43	0.45
Target lesion stent length (mm)	31 ± 10	31 ± 10	31 ± 10	0.94
More than one stent deployed	79 (30.4)	35 (26.7)	44 (34.1)	0.20
Total stent number in target artery (*n*)	1.4 ± 0.7	1.5 ± 0.7	1.4 ± 0.6	0.49
Total stent length in target artery (mm)	41 ± 20	42 ± 21	41 ± 19	0.67
Post-dilation of stent	255 (98.1)	130 (99.2)	125 (96.9)	0.17
Post-dilation balloon diameter (mm)	3.75 ± 0.58	3.72 ± 0.58	3.79 ± 0.58	0.33
Post-dilation pressure (atm)	17 ± 3	17 ± 3	17 ± 2	0.74
Diameter difference post-dilation balloon to stent	0.5 ± 0.4	0.5 ± 0.4	0.5 ± 0.4	0.63

Values are *n* (%) or mean ± SD.

ACS, acute coronary syndrome; IVUS, intravascular ultrasound; LAD, left anterior descending; LCx, left circumflex; MI, myocardial infarction; NS, non-significant; NSTE-ACS, non-ST-elevation acute coronary syndrome; NSTEMI, Non-ST-elevation myocardial infarction; OCT, optical coherence tomography; OM, obtuse marginal; PCI, percutaneous coronary intervention; PIOS, physiology-guided incremental optimization strategy; QCA, quantitative coronary angiography; RCA, right coronary artery; STEMI, ST-elevation myocardial infarction.

**Figure 3:**
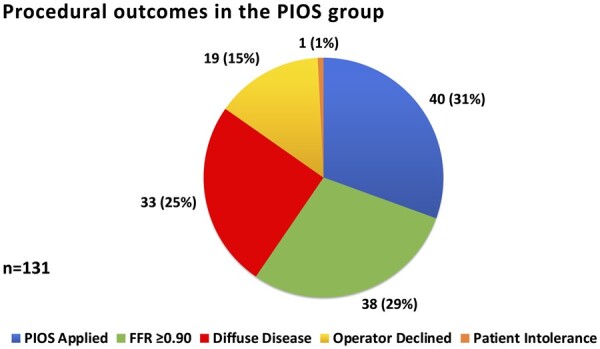
Procedural outcomes in the physiologically-guided incremental optimization strategy group. Following an initial post-percutaneous coronary intervention fractional flow reserve assessment, 29% of patients had a fractional flow reserve ≥0.90 and did not require optimization. Of the remaining 93 patients with fractional flow reserve <0.90, 33 had diffuse residual patterns, which did not meet the protocol-defined criteria for further intervention. Targets for additional intervention were identified in 60 patients. Operators attempted functional optimization in 40 of these patients. The remaining 20 cases in which optimization attempts were not undertaken are discussed in the Supplementary material online. FFR, fractional flow reserve; PIOS, physiologically guided incremental optimization strategy.

The mean initial post-PCI FFR for the overall population was 0.85 ± 0.09. Intracoronary imaging was utilized during the initial PCI in 16.2% (42/260) of patients. There was no significant difference in initial post-PCI FFR between those with ICI-guided PCI (0.83 ± 0.09) and those guided by angiography alone (0.85 ± 0.09, difference −0.02, 95% CI −0.05 to 0.01, *P* = 0.26). Overall, 30.5% (40/131) of patients randomized to the PIOS group received further intervention (*Figure 3*). The left anterior descending (LAD) artery was the target vessel in 85% (34/40) of these patients. There were no significant differences in physiology indices between randomized groups with respect to final mean FFR values or the absolute and relative changes from pre-PCI to final post-PCI phases (*Table 3*). 34/117 (29.1%) in the PIOS group had an initial post-PCI FFR ≤0.80, improving to 22/118 (18.6%) following additional FFR-guided optimization. The proportion of vessels with post-PCI FFR ≥0.90 in the PIOS group was 42/117 (35.9%) initially, increasing to 45/118 (38.1%) following additional PCI.

**Table 3:** Coronary physiology characteristics

		Total (260)				PIOS (131)				Control (129)				*P*-value
Index	Stage	*N* (%)	Value	Absolute change	Relative change (%)	*N* (%)	Value	Absolute change	Relative change (%)	*N* (%)	Value	Absolute change	Relative change (%)	
Pd/Pa	Pre	242 (93)	0.76 ± 0.18			126 (96)	0.78 ± 0.16			116 (90)	0.73 ± 0.19			
	Final	246 (95)	0.94 ± 0.05	0.18 ± 0.18	35 ± 55	122 (93)	0.94 ± 0.05	0.15 ± 0.16	27 ± 49	124 (96)	0.94 ± 0.06	0.21 ± 0.19	43 ± 60	0.98
dPR	Pre	242 (93)	0.70 ± 0.21			126 (96)	0.73 ± 0.20			116 (90)	0.67 ± 0.22			
	Final	246 (95)	0.92 ± 0.06	0.22 ± 0.20	54 ± 86	122 (93)	0.93 ± 0.06	0.19 ± 0.19	41 ± 66	124 (96)	0.92 ± 0.07	0.25 ± 0.22	67 ± 101	0.68
RFR	Pre	242 (93)	0.68 ± 0.23			126 (96)	0.71 ± 0.21			116 (90)	0.64 ± 0.24			
	Final	246 (95)	0.92 ± 0.06	0.24 ± 0.22	71 ± 132	122 (93)	0.92 ± 0.06	0.21 ± 0.20	51 ± 89	124 (96)	0.91 ± 0.07	0.28 ± 0.23	93 ± 163	0.38
TT rest	Pre	240 (92)	1.12 ± 0.45			121 (92)	1.12 ± 0.43			119 (92)	1.12 ± 0.47			
	Final	255 (98)	0.93 ± 0.42	−0.20 ± 0.45	−10 ± 43	127 (97)	0.94 ± 0.43	−0.21 ± 0.40	−13 ± 41	128 (99)	0.92 ± 0.42	−0.19 ± 0.50	−8 ± 44	0.37
TT hyp	Pre	236 (91)	0.67 ± 0.38			122 (93)	0.66 ± 0.36			114 (88)	0.69 ± 0.41			
	Final	255 (98)	0.32 ± 0.20	−0.36 ± 0.39	−41 ± 44	125 (95)	0.33 ± 0.20	−0.34 ± 0.36	−40 ± 46	127 (98)	0.33 ± 0.20	−0.38 ± 0.42	−42 ± 42	0.90
CFR	Pre	233 (90)	1.9 ± 0.9			120 (92)	2.0 ± 0.8			113 (88)	1.9 ± 1.0			
	Final	252 (97)	3.4 ± 1.9	1.5 ± 1.8	99 ± 127	125 (95)	3.4 ± 1.8	1.3 ± 1.7	84 ± 111	127 (98)	3.4 ± 2.0	1.6 ± 1.9	114 ± 140	0.12
IMR	Pre	223 (86)	28 ± 12			117 (89)	28 ± 12			106 (82)	27 ± 12			
	Final	248 (95)	22 ± 16	−7 ± 15	−14 ± 66	122 (93)	22 ± 16	−7 ± 16	−14 ± 74	126 (98)	21 ± 16	−7 ± 15	−15 ± 57	0.68
IMRc	Pre	223 (86)	21 ± 10			117 (89)	22 ± 11			106 (82)	19 ± 10			
	Final	248 (95)	21 ± 16	0 ± 14	14 ± 120	122 (93)	22 ± 16	−1 ± 15	12 ± 106	126 (98)	20 ± 16	0 ± 14	16 ± 133	0.81
FFR	Pre	236 (91)	0.59 ± 0.14			126 (96)	0.60 ± 0.14			110 (85)	0.57 ± 0.15			
	Final	239 (92)	0.86 ± 0.08	0.27 ± 0.15	56 ± 45	118 (90)	0.86 ± 0.08	0.25 ± 0.15	50 ± 40	121 (94)	0.85 ± 0.08	0.29 ± 0.15	63 ± 50	0.93

Values are *n* (%) or mean ± SD.

Pd/Pa, ratio of mean distal coronary to aortic pressure at rest; dPR, diastolic pressure ratio; RFR, resting full-cycle ratio; TT rest, mean resting transit time; TT hyp, mean hyperaemic transit time; CFR, coronary flow reserve; IMR, index of microcirculatory resistance; IMRc, index of microcirculatory resistance corrected for epicardial stenosis; FFR, fractional flow reserve; Pre, pre-PCI measurement; Final, post-PCI value at end of procedure; PIOS, physiology-guided incremental optimization strategy.

The primary and secondary endpoint results are presented in *Table 4*. There was no significant difference between groups with respect to the primary endpoint of the proportion of patients with final post-PCI FFR ≥0.90 (PIOS minus control 10%, 95% CI −1.84 to 21.91, *P* = 0.099). The proportion of patients with final FFR ≤0.80 was significantly lower in the PIOS group (PIOS minus control −11.2%, 95% CI −21.87 to −0.35, *P* = 0.045).

**Table 4.** Primary and secondary endpoints

	PIOS	Control	*P*-value
Primary endpoint			
Final FFR ≥0.90 (%)			
Patients analysed (*n*)	118	121	
Proportion ≥0.90 (%)	38.1	28.1	
Difference between groups (95% CI)	10 [−1.84 to 21.91]		0.099
Secondary endpoints			
Final FFR ≤0.80 (%)			
Patients analysed (*n*)	118	121	
Proportion ≤0.80 (%)	18.6	29.8	
Difference between groups (95% CI)	−11.2 [−21.87 to −0.35]		0.045
Final dPR ≥0.90 (%)			
Patients analysed (*n*)	122	126	
Proportion ≥0.90 (%)	63.9	65.1	
Difference between groups (95% CI)	−1.2 [−13.1 to 10.8]		0.85
Final RFR ≥0.90 (%)			
Patients analysed (*n*)	122	126	
Proportion ≥0.90 (%)	59	60.3	
Difference between groups (95% CI)	−1.3 [−13.5 to 13.9]		0.83
Final CFR ≥2.0 (%)			
Patients analysed (*n*)	125	127	
Proportion ≥2.0 (%)	78.4	78	
Difference between groups (95% CI)	0.4 [−9.8 to 10.7]		0.93
Final IMR ≥25			
Patients analysed (*n*)	122	126	
Proportion ≥25 (%)	26.2	21.4	
Difference between groups (95% CI)	4.8 [−5.8 to 15.4]		0.37
Final IMRc ≥25			
Patients analysed (*n*)	122	126	
Proportion >25 (%)	24.6	19.8	
Difference between groups (95% CI)	4.8 [−5.6 to 15.1]		0.37
Change in SAQ summary score			
Patients analysed (*n*)	114	115	
Change between pre-PCI and 3-month follow-up scores	20.95 ± 25.04	21.51 ± 24.55	
Difference between groups (95% CI)	−0.56 [−5.9 to 7.0]		0.68
Change EQ-5D-5L			
Patients analysed (*n*)	114	114	
Change between pre-PCI and 3-month follow-up scores	0.06 ± 0.22	0.03 ± 0.21	
Difference between groups (95% CI)	0.03 [−0.03 to 0.08]		0.64
Target vessel failure			
Target vessel failure (*n*)	1	0	
Cardiac death	1	0	
Target vessel myocardial infarction	0	0	
Target vessel revascularization	0	0	

CFR, coronary flow reserve;dPR, diastolic pressure ratio; EQ-5D-5L, 5-level version of EuroQol-5 Dimension questionnaire; FFR, fractional flow reserve; IMR, index of microcirculatory resistance; IMRc, index of microcirculatory resistance corrected for epicardial stenosis; PIOS, physiology-guided incremental optimization strategy; RFR, resting full-cycle ratio; SAQ, Seattle Angina Questionnaire; TT hyp, mean hyperaemic transit time; TT rest, mean resting transit time.

A per-protocol procedural and safety analysis of patients in the PIOS group who underwent additional optimization identified that this group had longer procedure durations with higher radiation, contrast and adenosine doses. There were no differences in the incidence of procedural complications when compared with those patients who did not receive additional interventions (Supplementary material online, *Table S1*).

Among patients who received further optimization, both FFR and CFR increased significantly. Patients receiving an additional stent had a greater increase in FFR than those who received further post-dilation alone (Supplementary material online, *Table S2*). Non-ST-elevation ACS patients were medically stabilized and underwent PCI on a priority outpatient basis at a median of 3 weeks following their ACS presentation. Pre-PCI FFR values were lower in this cohort but there was no significant difference in microvascular resistance (as represented by corrected IMR) compared with patients undergoing PCI for either stable angina or staged completion of revascularization in non-culprit vessels (Supplementary material online, *Table S3*). When stratified by target vessel, there was no difference in mean pre-PCI FFR values between vessels, however, post-PCI FFR was significantly lower in the LAD (Supplementary material online, *Table S4*). The LAD had a lower proportion of patients with a final FFR ≥0.90 and a higher proportion with FFR ≤0.80 when compared with other vessels (Supplementary material online, *Table S5*).

The relative (percentage) change in FFR following PCI had a moderate but significant correlation with SAQ angina frequency score at 3-month follow-up (Spearman correlation coefficient 0.36, *P* < 0.0001). Larger relative increases in FFR were associated with a reduced burden of patient-reported angina at follow-up (*Table 5*). There were no peri-procedural deaths. At a median follow-up of 2 years, there had been only one target vessel failure. This patient, who was in the PIOS group but had not received any additional optimization, suffered a presumed cardiac death in the community 17 months after the procedure.

**Table 5:** Follow-up Seattle Angina Questionnaire scores stratified by tertiles of relative (percentage) change in FFR among patients with angina at baseline

SAQ domain	Low FFR Change		Intermediate FFR Change		High FFR Change		*P*-value
	*N*	Score	*N*	Score	*N*	Score	
Physical Limitation Score	47	71.81 ± 30.07	48	83.16 ± 24.76	52	85.90 ± 23.89	0.02
Angina Frequency Score	50	78.60 ± 23.56	55	85.09 ± 21.33	57	94.39 ± 13.89	<0.001
Quality of Life Score	50	71.75 ± 29.75	55	77.95 ± 28.05	57	84.43 ± 23.89	0.06
SAQ Summary Score	50	74.51 ± 24.20	55	81.45 ± 22.38	57	88.23 ± 18.29	0.01

## Discussion

In this randomized controlled trial of a coronary physiology-guided PCI optimization strategy, additional FFR-guided intervention after stenting did not significantly increase the proportion of patients achieving the optimal post-PCI FFR result of ≥0.90, but did reduce the proportion of patients with a final FFR ≤0.80 (the guideline-directed threshold for revascularization) when compared with the angiography-guided control group (*Graphical abstract*)

There is a gap in clinical trial evidence on strategies to optimize PCI outcomes.^29^ In a retrospective, single-centre registry of 664 vessels from 574 patients, 118 (17.8%) vessels were found to have a post-PCI FFR ≤0.80.^19^ Additional interventions were performed in 87/118 (73.7%) of these vessels which increased the final FFR to ≥0.80 in 58/87 (66.7%). In the overall population, additional post-dilation or stenting reduced the proportion of vessels with post-PCI FFR ≤0.80 from 118 (17.8%) to 63 (9.5%). In total, 137 vessels (20.6%) underwent further treatment for what were perceived to be suboptimal post-PCI FFR results with further post-dilatation (42%) and/or additional stenting (33%) or both (18%). Fractional flow reserve was repeated in all 137 lesions with an overall improvement from 0.78 ± 0.07 to 0.87 ± 0.05. Amongst patients who received post-dilatation only, FFR improved from 0.75 ± 0.06 to 0.85 ± 0.06. This is perhaps not surprising when the particulars of the index PCI procedure are examined. Just over half of lesions (*n* = 352, 53%) were pre-dilated, the mean diameter of implanted stents was 2.87 mm and only 200 (30.1% of vessels) received post-dilatation. By comparison, in TARGET-FFR, the mean stent diameter was 3.23 mm with 100% and 98.1% rates of pre-dilatation and post-dilatation (with on average a 0.5 mm larger non-compliant balloon), respectively (*Table 2*). The yield from additional post-dilatation alone in our study was more modest (FFR increased from 0.79 ± 0.07 to 0.83 ± 0.05, Supplementary material online, *Table S2*) and could suggest a higher incidence of initial stent under-expansion and/or malapposition in the previous registry.

A recent prospective registry supports the findings from our trial.^14^ In this registry, 84/230 vessels (36.5%) had an initial post-PCI FFR ≤0.80 while just 49/230 (21.3%) achieved a value >0.90. Fractional flow reserve pullback identified targets for further optimization in 29/84 (34.5%). After further intervention, FFR increased from 0.73 (interquartile range: 0.69–0.77) to 0.80 (interquartile range: 0.77–0.85) and reduced the overall incidence of post-PCI FFR ≤0.80 by 6.1% (36.5–30.4%). The number of vessels with a final FFR >0.90 increased by 5 (2.1%). In TARGET-FFR’s PIOS group, additional FFR-guided optimization measures reduced the overall incidence of FFR ≤0.80 by 10.5% and increased the proportion of vessels with post-PCI FFR ≥0.90 by 2.2%.

Why is it so difficult to achieve a post-PCI FFR ≥0.90? The answer may be influenced by characteristics of the target coronary artery. Percutaneous coronary intervention on a lesion in the LAD has previously been identified as an independent predictor of suboptimal post-PCI FFR results^14^ ^,^ ^19^ ^,^ ^21^ ^,^ ^22^ ^,^ ^30^ and the LAD was the target vessel in 150/260 (57.7%) of patients in TARGET-FFR. Our data confirm that both absolute post-PCI FFR values and the proportion of patients achieving a final FFR value ≥0.90 were significantly lower in the LAD than either the left circumflex or right coronary arteries. There were, however, no significant differences between vessels in post-PCI CFR or corrected microvascular resistance. It has been postulated that lower FFR values in the LAD relate to the larger area of myocardium subtended by this vessel. Higher flow rates across long segments of residual mild diffuse atheroma can result in large pressure gradients in these vessels. Hydrostatic factors relating to coronary anatomy and the height of the pressure wire sensor above or below the aortic pressure transducer may also contribute to this phenomenon. Given that achieving a post-PCI FFR ≥0.90 in the LAD in anything other than a minority of patients appears unlikely, does this threshold actually represent a realistic target or definition of a functionally optimal PCI result in this vessel? In a registry of 835 patients who had post-PCI FFR measured, Hwang *et al.* ^23^ reported that the optimal cut-off values for predicting target vessel failure at 2 years were lower in LAD than in non-LAD vessels (0.82 vs. 0.88).

In the present study, use of ICI during the index PCI was not associated with higher post-PCI FFR values when compared with cases guided by angiography alone. Intravascular ultrasound (IVUS) or optical coherence tomography (OCT) was utilized in 16.2% (42/260) of PCI procedures, which exceeds the UK national average of 10.7% in 2018/19. The DOCTORS study previously randomized 240 patients with non-ST-elevation ACS to either OCT- or angiography-guided PCI and reported a marginally higher post-PCI FFR in the OCT arm (0.94 ± 0.04 vs. 0.92 ± 0.05, *P* = 0.005).^31^ It is worth noting that observational data from a number of non-randomized studies in which IVUS was routinely used before and after PCI^7^ ^,^ ^11^ ^,^ ^18^ have reported mean/median post-PCI FFR values within the same range as those from cohorts with lower rates of adjunctive ICI than in TARGET-FFR.^9^ ^,^ ^13^ ^,^ ^25^

Even well-expanded stents manifest a pressure gradient during maximal hyperaemia. In TARGET-FFR, we theorized that a trans-stent gradient of >0.05 FFR units would be of sufficient magnitude to actually detect change/improvement related to additional post-dilation of the stent. Yang *et al.* ^32^ have reported that trans-stent gradients ≥0.04 FFR units are associated with increased rates of MACE. The authors found that despite successful, IVUS-assessed PCI, 98.5% of stents in their study had a HTG >0, with single stents having a mean HTG of 0.03 ± 0.02 and overlapping stents a mean of 0.05 ± 0.02. These findings support our hypothesis that intervening on stents with HTG <0.05 units would have been unlikely to achieve an appreciable change in final FFR.

Currently, there are no generally accepted definitions of focal or diffuse disease with respect to pressure-wire pullback curves. To date, such definitions have been arbitrary, vary from study to study, and are often very much in the eye of the beholder. The DEFINE PCI study examined post-PCI instantaneous wave-free ratio (iFR) pullbacks and arbitrarily categorized trans-stenotic pressure gradients ≥0.03 iFR units as focal lesions when their length was ≤15 mm, and as diffuse disease when their length exceeded 15 mm.^33^ Fifteen millimetres is not an insignificant length of vessel and a plausible argument could be made that a relatively small pressure loss (0.03 units) over such length should be considered diffuse. Adopting such a broad definition of focality led the authors to the unprecedented conclusion that 81.6% of patients with post-PCI iFR <0.90 had ‘focal’ residual disease. The signal to noise ratio is lower with hyperaemic pullbacks than with resting assessments. Had we chosen to define focal disease as a gradient of ≥0.03 FFR units over 15 mm, the data from Yang *et al.* illustrate how a pre-PCI gradient of 0.03 within a vessel could end up being replaced by an HTG of 0.03 post-stenting. This would achieve no overall functional gain in the vessel yet expose the patient to the risk of additional coronary intervention. As one can observe from the case examples provided in the Supplementary material online, truly focal lesions cause an obvious and abrupt drop in pressure. Accordingly, we felt that an abrupt pressure drop ≥0.05 FFR units was an appropriate definition of focal disease when utilizing FFR assessment (with anything else being considered generally diffuse).

TARGET-FFR provides the first randomized data on the incidence of physiologically suboptimal results following standard-of-care PCI and confirms the feasibility of routine post-PCI FFR assessment. It found that persistently abnormal post-PCI FFR values are common and that a strategy of routine post-PCI physiology guidance can safely and effectively improve the final FFR values in a significant number of the worst-affected patients. Importantly, larger relative increases in FFR were associated with a reduced frequency of angina at 3-month follow-up.

The trial has a number of limitations. It is a single-centre study with a relatively homogeneous PCI practice, including high rates of lesion pre-dilatation and high-pressure stent post-dilatation. On average, FFR is performed prior to PCI in 9.4% of cases in the UK. In our study, the rate of pre-PCI FFR guidance (including pullback assessment) was 35%. This may have influenced or altered operators’ stenting strategy and consequently reduced the incidence of focal, physiologically significant residual disease post-PCI. A larger multicentre trial incorporating a wider range of PCI strategies and techniques may have had a different outcome. With just 40 of the 131 patients randomized to the PIOS arm actually receiving additional optimization measures, the study was ultimately underpowered to detect a significant between-group difference for its primary endpoint. Excluding patients with post-PCI FFR ≥0.90 (29% of the PIOS group) from randomization would have increased the power for the primary endpoint but overestimated the effect physicians could expect from measuring post-PCI physiology. Ultimately, TARGET-FFR was a trial of a strategy of routine post-PCI FFR assessment vs. standard of care, without selection based on the post-PCI FFR value. This approach permits an evaluation of the effects of the PIOS intervention, regardless of the baseline post-PCI FFR. By randomizing all-comers, the design allowed a comprehensive, and generalizable evaluation of the effects of physiology-guided PCI optimization. The incidence of target vessel failure at a median follow-up of 2 years was very low and the study was not powered for clinical outcomes. Larger randomized trials would be required to test if physiology-guided optimization of PCI results can improve patient outcomes compared with standard angiographic assessment alone.

## Supplementary material

Supplementary material is available at *European Heart Journal* online.

## Supplementary Material

ehab449_Supplementary_Material
