# Supplementary material for: Post-stenting fractional flow reserve vs coronary angiography for optimization of percutaneous coronary intervention (TARGET-FFR)
Source: Eur Heart J. 2021 Jul 19;42(45):4656–68. doi: 10.1093/eurheartj/ehab449 (PMC8634564; doi:10.1093/eurheartj/ehab449)
Supplement: ehab449_Supplementary_Material [file ehab449_supplementary_material.docx]

**Supplementary Material**

**Physiology core lab procedures**

All pressure tracings were sent to the CoreAalst (Aalst, Belgium) Physiology Core Laboratory for independent review and adjudication.

Analyses were performed following internal standard operational procedures using CoroFlow version 3.5 software (Coroventis research, Uppsala, Sweden). Each individual tracing was assessed for quality based on pre-specified criteria. Each tracing received a binary decision regarding adequate quality for inclusion and FFR was calculated independently for each tracing.

Aortic pressure tracings were visually adjudicated based on the quality of the aortic pressure waveform defined by the following criteria:

1. Presence of dicrotic notch.
2. Absence of ventricularization
3. Absence of pressure curve distortion
4. Absence of arrhythmia
5. Stability of hyperaemia

Fractional flow reserve (FFR) was defined as the lowest value during steady state hyperemia. Drift was evaluated when available and reported as a continuous variable. FFR measurements with drift greater than 0.03 FFR units were excluded.

Temperature/pressure tracings were adjudicated based on completeness of measurement and morphology of the temperature waveform. In addition, stability of the intracoronary pressure tracing (Pd) was also assessed as this influences IMR calculation.

Temperature tracings were quantitively evaluated to extract the following parameters:

1. Maximal temperature reduction (MinTemp): mean of maximal temperature reduction of three saline injections
2. Temperature decrease time (TDT): Time from the beginning of the temperature reduction to nadir temperature.
3. Transit recovery time (TRT): duration (seconds) from the nadir of the hyperaemic thermodilution curve in the distal sensor to 20% from baseline temperature.

Tracings (i.e. temperature and accompanying pressure tracings) were visually adjudicated and considered adequate for analysis based on the following criteria:

1. Absence of major coronary pressure waveform artefact (defined as cyclic oscillation of the pressure curve with change in Pd more than 10mmHg).
2. Presence of Pa curve.
3. Complete set of injections.

For the IMR calculation, the Pd was selected at the level of the lowest fractional flow reserve (FFR). Higher than expected variability in Tmn identified by the Coroflow software was recorded but this was not considered a reason for exclusion of the measurement.

**Supplementary Table 1. Per-protocol analysis of procedural characteristics and complications**

|  | **PIOS (40)** | **No PIOS (220)** | **p value** |
| --- | --- | --- | --- |
| **Procedural characteristics** |  |  |  |
| Procedure duration (mins) | 94±23 | 67±24 | <0.0001 |
| Total contrast dose (ml) | 225±53 | 185±51 | <0.0001 |
| Fluoroscopy time (mins) | 23±8 | 16±8 | <0.0001 |
| Dose Area Product (cGy.cm^2^) | 5236±2783 | 3780±2391 | 0.0007 |
| Radiation dose (mGy) | 921±551 | 686±462 | 0.0043 |
| Total duration of adenosine infusions (sec) | 439±87 | 290±73 | <0.0001 |
| Total adenosine dose (mg) | 93±25 | 62±32 | <0.0001 |
|  |  |  |  |
| **Procedural complications** |  |  |  |
| Coronary dissection | 0 | 2 (0.9%) | 0.54 |
| Side branch occlusion | 1 (2.5%) | 8 (3.6%) | 0.72 |
| No flow / slow flow phenomenon | 0 | 2 (0.9%) | 0.54 |
| Arm haematoma >5cm | 0 | 10 (4.5%) | 0.17 |
| Type 4a myocardial infarction | 0 | 7 (3.2%) | 0.60 |

Procedure time and doses of contrast, radiation and adenosine were significantly higher among patients who received additional optimisation measures. There were no significant differences in the incidence of procedural complications between groups.

**Supplementary Table 2. Physiological effects of the PIOS intervention**

|  | **Initial Post-PCI** | | **Final Post-PCI** | | **Absolute Difference** | | **Relative Difference (%)** | | **p value** |
| --- | --- | --- | --- | --- | --- | --- | --- | --- | --- |
|  | N | Value | N | Value | N | Value | N | Value |  |
| **Any PIOS received (40)** |  |  |  |  |  |  |  |  |  |
| FFR | 33 | 0.76±0.08 | 34 | 0.82±0.06 | 29 | 0.06±0.07 | 29 | 9±11.7 | <0.0001 |
| CFR | 40 | 3.0±1.6 | 35 | 4.0±2.1 | 35 | 1.0±2.2 | 35 | 56.7±103.7 | 0.02 |
| IMR | 39 | 20±7 | 33 | 18±7 | 33 | -3±8 | 33 | -6.2±38.3 | 0.08 |
| IMRc | 39 | 19±7 | 33 | 17±7 | 33 | -2±8 | 33 | -3.2±41.3 | 0.17 |
| **Balloon only (23)** |  |  |  |  |  |  |  |  |  |
| FFR | 18 | 0.79±0.07 | 19 | 0.83±0.05 | 15 | 0.03±0.05 | 15 | 4.4±7.6 | 0.03 |
| CFR | 23 | 3.1±1.6 | 19 | 3.6±1.5 | 19 | 0.4±1.4 | 19 | 32.9±62.1 | 0.21 |
| IMR | 22 | 19±6 | 17 | 16±6 | 17 | -3±7 | 17 | -8.9±34.1 | 0.11 |
| IMRc | 22 | 18±6 | 17 | 15±5 | 17 | -2±7 | 17 | -7.3±36.2 | 0.17 |
| **Stent only (12)** |  |  |  |  |  |  |  |  |  |
| FFR | 11 | 0.72±0.09 | 10 | 0.80±0.05 | 10 | 0.09±0.08 | 10 | 14.3±15.8 | 0.01 |
| CFR | 12 | 2.8±1.8 | 11 | 5.4±2.6 | 11 | 2.5±2.8 | 11 | 127.1±142.8 | 0.01 |
| IMR | 12 | 24±9 | 11 | 18±10 | 11 | -6±10 | 11 | -19.6±35.3 | 0.10 |
| IMRc | 12 | 22±8 | 11 | 17±10 | 11 | -4±10 | 11 | -15.6±37.9 | 0.19 |
| **Post-dilation and stent (5)** |  |  |  |  |  |  |  |  |  |
| FFR | 4 | 0.75±0.04 | 5 | 0.85±0.07 | 4 | 0.10±0.05 | 4 | 12.8±6.9 | 0.04 |
| CFR | 5 | 2.9±1.2 | 5 | 2.4±1.2 | 5 | -0.5±1.6 | 5 | -7.5±40 | 0.54 |
| IMR | 5 | 16±5 | 5 | 20±6 | 5 | 4±4 | 5 | 32.1±39.9 | 0.09 |
| IMRc | 5 | 15±5 | 5 | 20±6 | 5 | 5±5 | 5 | 38±47.1 | 0.09 |

Values are n (%) or mean±SD. CFR=Coronary Flow Reserve; IMR=Index of Microcirculatory Resistance; IMRc= Index of Microcirculatory Resistance corrected for epicardial stenosis; FFR=Fractional Flow Reserve; PCI=Percutaneous Coronary Intervention; PIOS=Physiology-guided Incremental Optimisation Strategy.

**Supplementary Table 3. Physiology stratified by PCI indication**

|  | **NSTEMI**  **(N=104)** | | **Stable Angina**  **(N=88)** | | **Staged Non-culprit**  **(N=68)** | | **p value** |
| --- | --- | --- | --- | --- | --- | --- | --- |
|  | N | Value | N | Value | N | Value |  |
| **Pre-PCI** |  |  |  |  |  |  |  |
| FFR | 92 | 0.55±0.15 | 79 | 0.57±0.14 | 65 | 0.67±0.10 | <0.0001 |
| CFR | 90 | 1.9±0.9 | 80 | 1.8±0.9 | 63 | 2.3±0.9 | 0.005 |
| IMR | 86 | 29±13 | 76 | 28±12 | 61 | 24±11 | 0.02 |
| IMRc | 86 | 21±11 | 76 | 21±9 | 61 | 20±10 | 0.98 |
| **Initial Post-PCI** |  |  |  |  |  |  |  |
| FFR | 97 | 0.86±0.10 | 80 | 0.83±0.08 | 61 | 0.85±0.09 | 0.11 |
| CFR | 104 | 3.3±1.7 | 87 | 3.5±2.1 | 66 | 2.9±1.5 | 0.15 |
| IMR | 103 | 23±17 | 85 | 19±11 | 66 | 24±19 | 0.13 |
| IMRc | 103 | 22±17 | 85 | 19±11 | 66 | 23±19 | 0.13 |

Values are n (%) or mean±SD. Pd/Pa=Ratio of mean distal coronary to aortic pressure at rest; dPR=diastolic pressure ratio; iwFR=instantaneous wave-free ratio; RFR=Resting Full-cycle Ratio; FFR=Fractional Flow Reserve; CFR=Coronary Flow Reserve; IMR=Index of Microcirculatory Resistance; IMRc= Index of Microcirculatory Resistance corrected for epicardial stenosis; PCI=Percutaneous Coronary Intervention; NSTEMI=Non-ST-segment-Elevation Myocardial Infarction.

**Supplementary Table 4. Physiology stratified by target vessel**

|  | **LAD**  **(N=150)** | | **LCx**  **(N=43)** | | **RCA**  **(N=67)** | | **p value** |
| --- | --- | --- | --- | --- | --- | --- | --- |
|  | N | Value | N | Value | N | Value |  |
| **Pre-PCI** |  |  |  |  |  |  |  |
| FFR | 135 | 0.58±0.14 | 39 | 0.61±0.11 | 62 | 0.59±0.16 | 0.52 |
| CFR | 132 | 2.1±1.1 | 41 | 1.8±0.8 | 60 | 1.8±0.6 | 0.06 |
| IMR | 131 | 26±10 | 38 | 27±13 | 54 | 32±15 | 0.02 |
| IMRc | 131 | 19±8 | 38 | 21±10 | 54 | 24±13 | 0.004 |
| **Initial Post-PCI** |  |  |  |  |  |  |  |
| FFR | 140 | 0.80±0.07 | 38 | 0.92±0.07 | 60 | 0.91±0.07 | <0.0001 |
| CFR | 148 | 3.2±1.8 | 43 | 3.3±1.4 | 66 | 3.4±2.1 | 0.82 |
| IMR | 146 | 22±15 | 43 | 19±11 | 65 | 25±19 | 0.19 |
| IMRc | 146 | 21±15 | 43 | 19±11 | 65 | 25±19 | 0.14 |

Values are n (%) or mean±SD. FFR=Fractional Flow Reserve; CFR=Coronary Flow Reserve; IMR=Index of Microcirculatory Resistance; IMRc= Index of Microcirculatory Resistance corrected for epicardial stenosis; PCI=Percutaneous Coronary Intervention; LAD=Left Anterior Descending artery; LCx=Left Circumflex artery; RCA=Right Coronary Artery

**Supplementary Table 5. Proportions of optimal (≥0.90) and suboptimal (≤0.80) final post-PCI FFR results stratified by target vessel**

|  | **LAD (N=138)** | **LCx (N=39)** | **RCA (N=62)** | **p value** |
| --- | --- | --- | --- | --- |
| **Final FFR ≥0.90** | 10 (7.2%) | 29 (74.4%) | 40 (64.5%) | <0.0001 |
| **Final FFR ≤0.80** | 52 (37.7%) | 2 (5.1%) | 4 (6.5%) | <0.0001 |

FFR=Fractional Flow Reserve; PCI=Percutaneous Coronary Intervention; LAD=Left Anterior Descending artery; LCx=Left Circumflex artery; RCA=Right Coronary Arter

**Protocol Deviations**

The following section contains summaries of all 20 cases in the PIOS group where additional optimisation measures were advised by the study protocol but were not performed due to operator and/or patient factors.

The pullback recordings also serve to provide examples of predominantly diffuse (but occasionally focal) residual disease patterns and some of the pressure waveform artefacts encountered during pullback assessments.

| 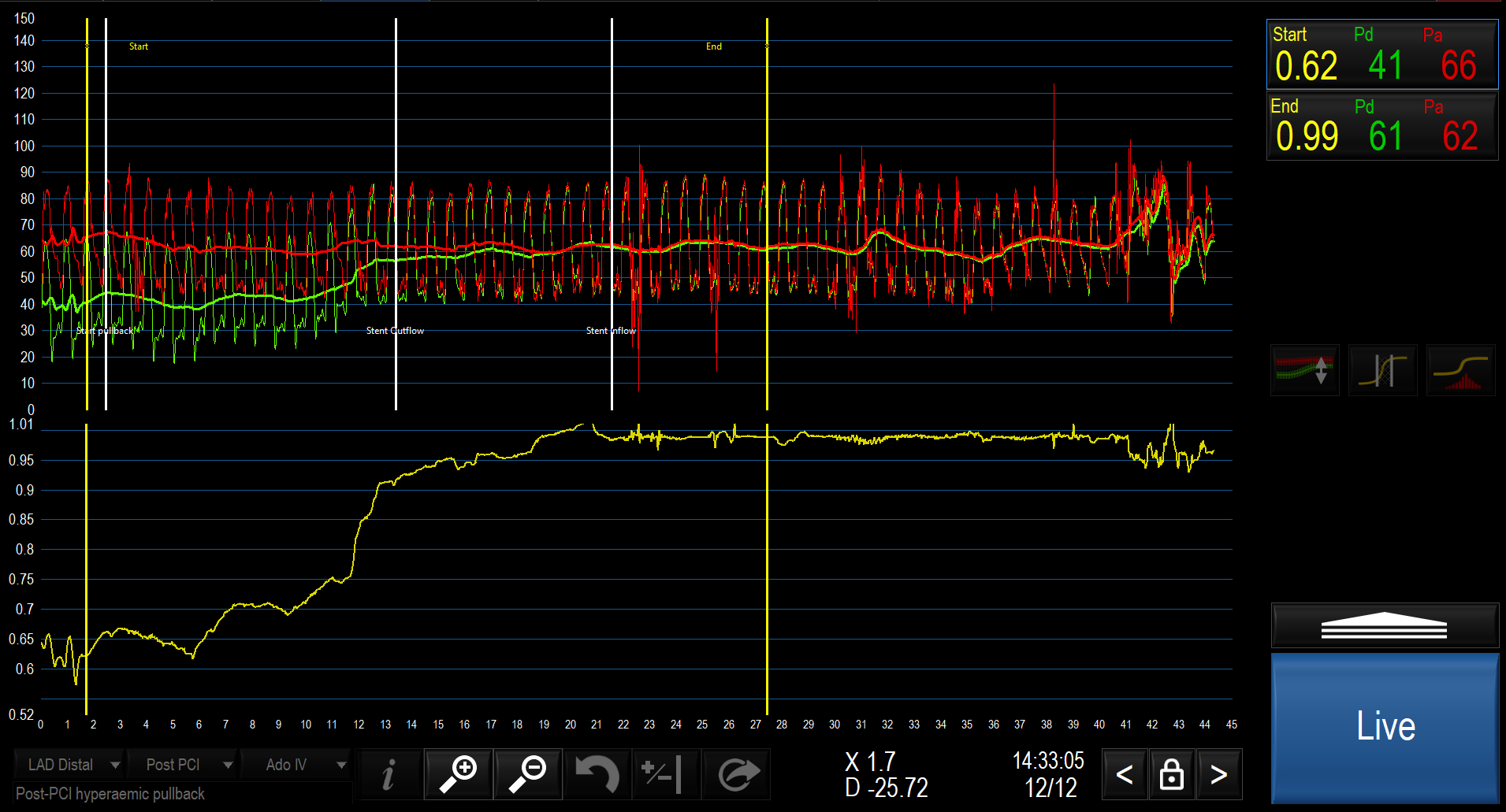 |
| --- |
| **Case 1.** Post-PCI FFR 0.61. A 3.0/33mm stent, post-dilated with 4mm non-compliant (NC) balloon to 18atm proximally. Diffuse residual gradient with hyperaemic trans-stent gradient (HTG) of 0.07 units and a focal pressure loss of 0.16 units at distal stent edge. Largest pressure drop occurred at the stent outflow in the mid LAD at the edge of segment with marked intramyocardial bridging. Operator felt stenting into the intramyocardial segment would be inappropriate and potentially hazardous |

| 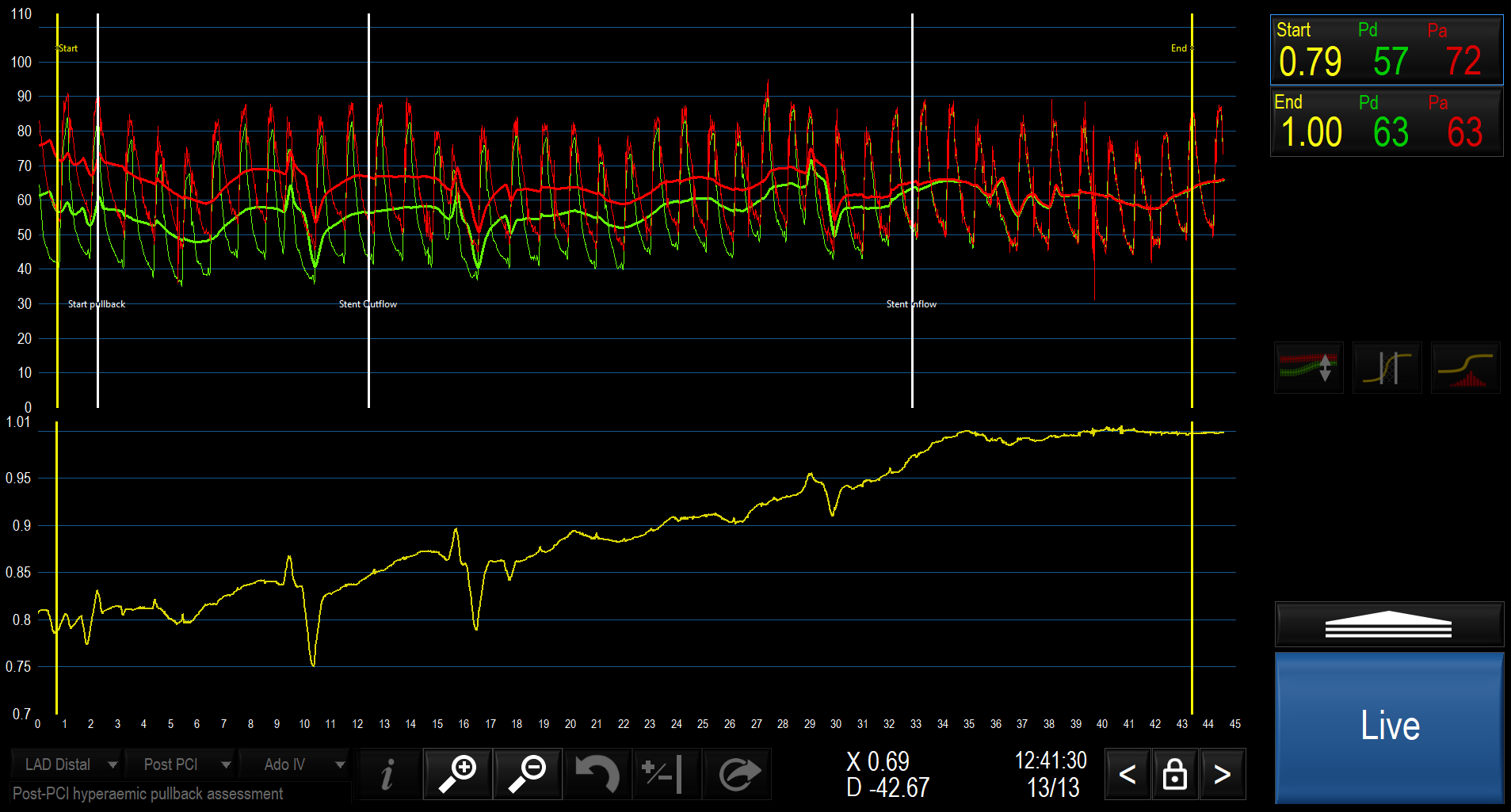 |
| --- |
| **Case 2.** Post-PCI FFR 0.78. Overlapping 3.0/32mm and 3.5/32mm stents post-dilated with 3.5mm NC balloon to 18atm. Diffuse residual gradient with HTG of 0.12 units. Operator felt sub-optimal post-PCI represented diffuse gradient in distal vessel and through the stented segment with no focus for additional optimisation following initial high pressure post-dilation with non-compliant balloons. Note pressure waveform artefacts from ectopic heartbeats. |

| 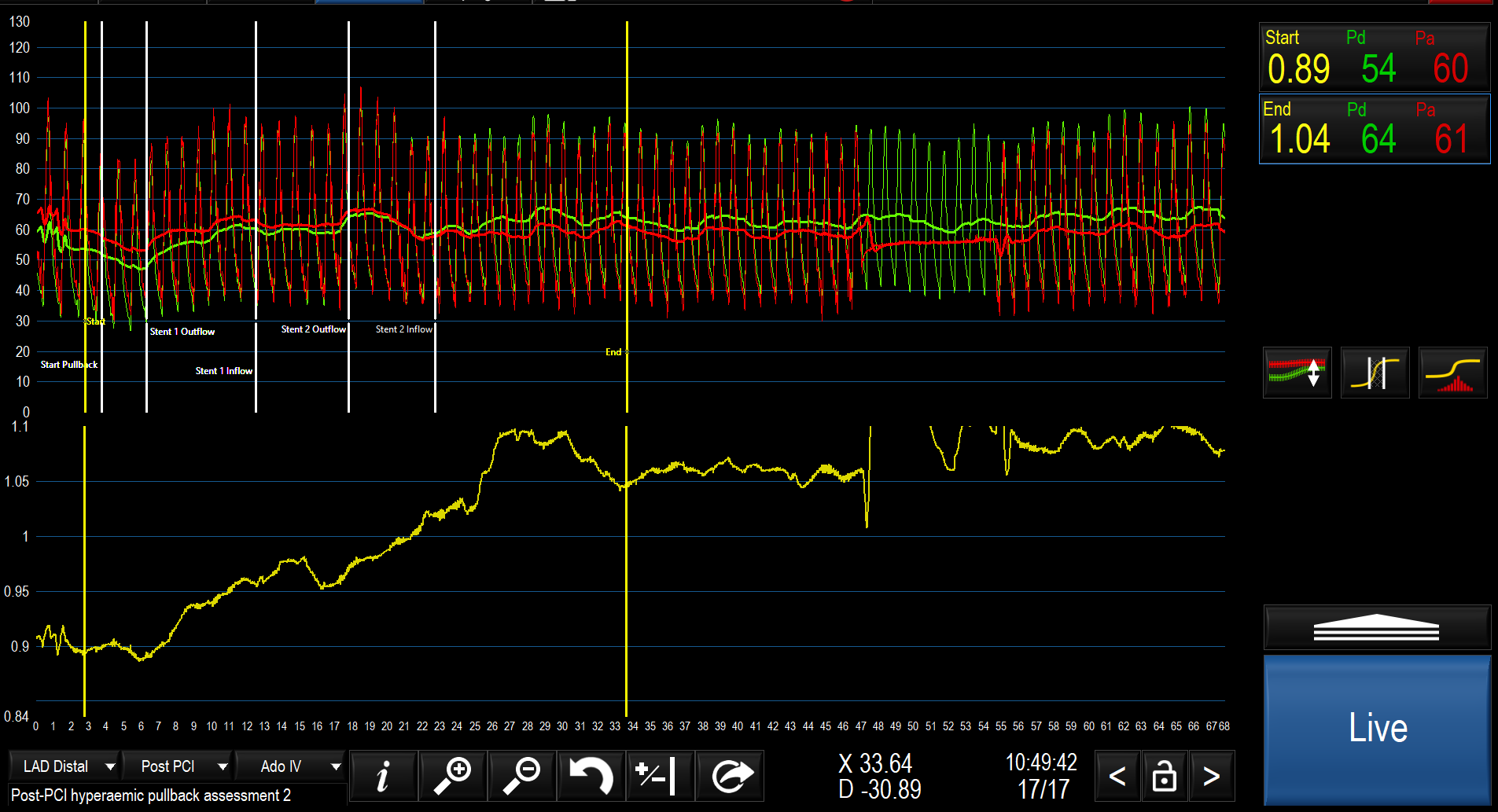 |
| --- |
| **Case 3.** Post-PCI FFR 0.88. 2.75/18mm stent deployed to mid vessel lesion, post-dilated with 3mm NC balloon to 16atm. 3.0/28mm stent to proximal lesion, post-dilated with 3.5mm NC balloon (14-18atm). HTG of 0.07 units across distal stent and 0.05 across proximal stent. Operator felt there were no further targets for optimisation and that the distal stent had been adequately post-dilated already |

| 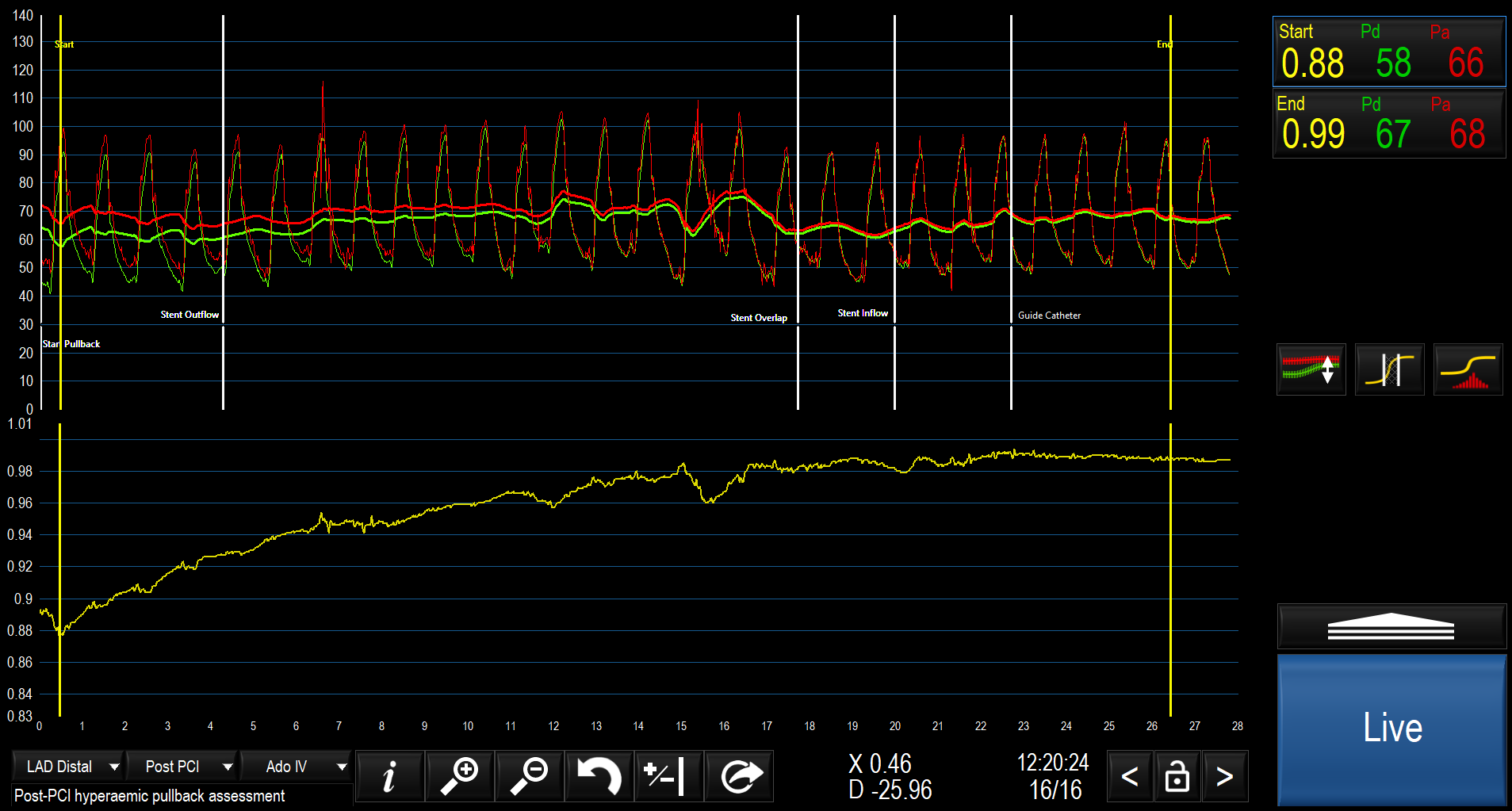 |
| --- |
| **Case 4.** Post-PCI FFR 0.87. Previous proximal LAD stent. New 3.0/32mm stent deployed to overlap distally, post-dilated with 3.5mm NC balloon to 22atm. Diffuse gradient with HTG of 0.05 units. Long stented segment with borderline HTG value. Operator felt further aggressive post-dilation had potential for an adverse outcome and declined to attempt further optimisation.. |

| 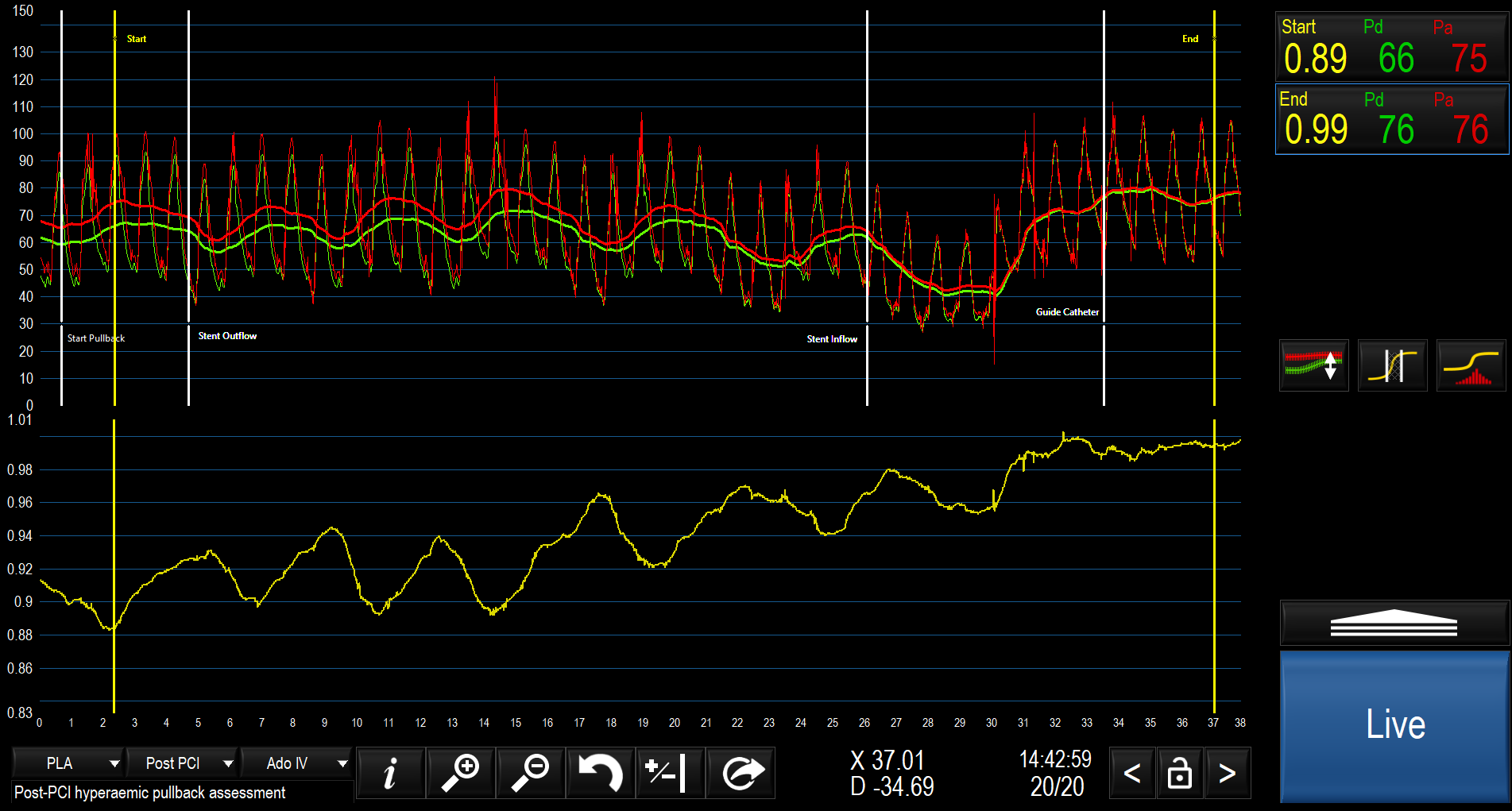 |
| --- |
| **Case 5.** Post-PCI FFR 0.86. Borderline HTG of 0.05 units across a 2.75/48mm stent which had been post-dilated with a 3mm NC balloon to 20atm. Operator felt appropriate post-dilation had been performed and that residual diffuse gradient related to stent length. Note undulating pressure waveform artefact related to pattern of adenosine response. |

| 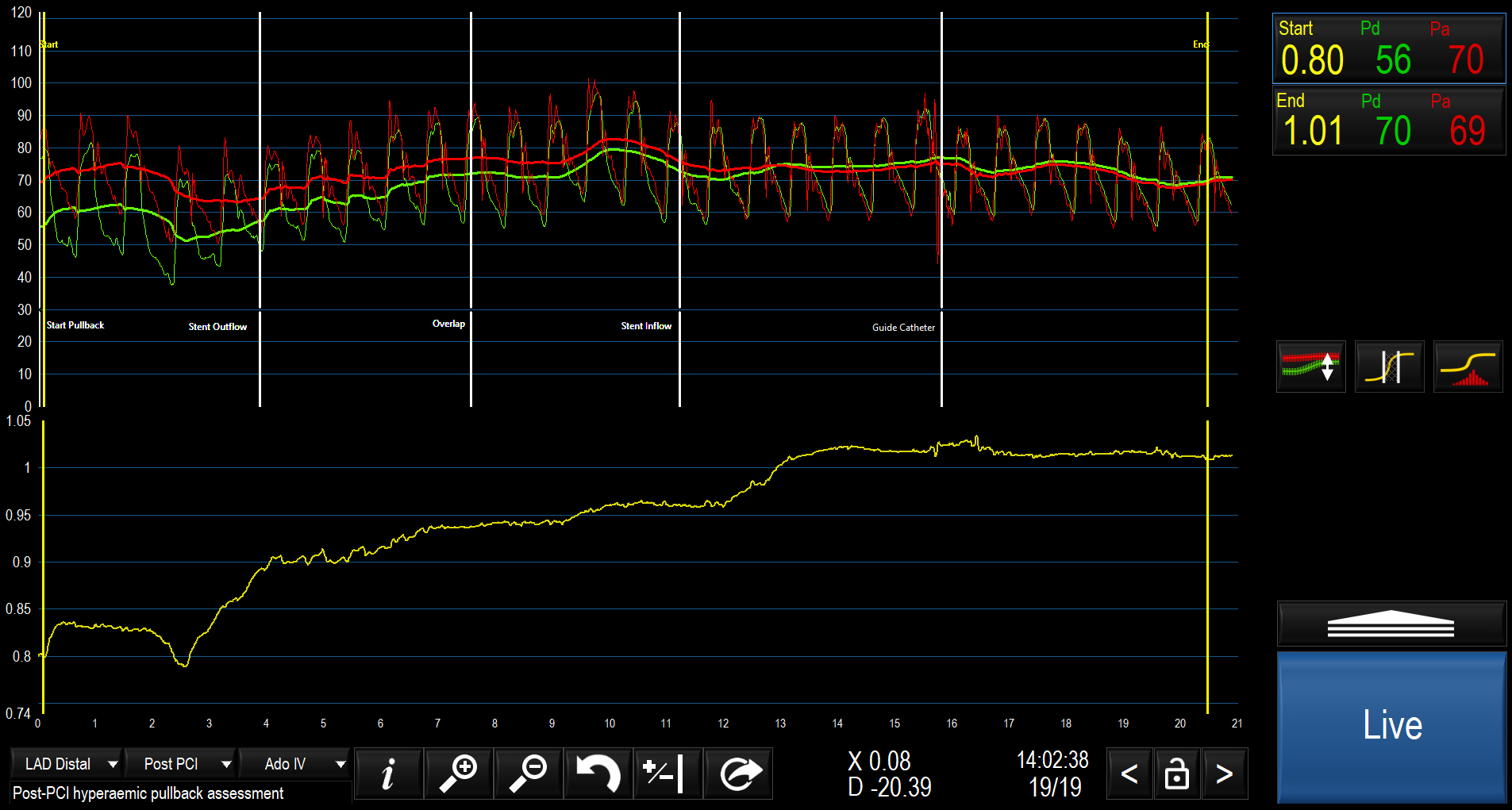 |
| --- |
| **Case 6.** Post-PCI FFR 0.80. 3.0/23mm stent post-dilated with 3.25mm NC balloon at target lesion. Additional 3.5/23mm stent overlapping proximally to cover inflow disease. Diffuse residual gradient with HTG of 0.07 units and a relatively focal drop of 0.10 units at stent outflow. Operator unwilling to perform further optimisation/post-dilate proximal stent as deployment had resulted in acute closure of a small, diseased septal branch which provoked seemingly disproportionate ischaemic chest pain and ECG changes (ST elevation in I and AVL, downsloping inferior ST depression) |

| 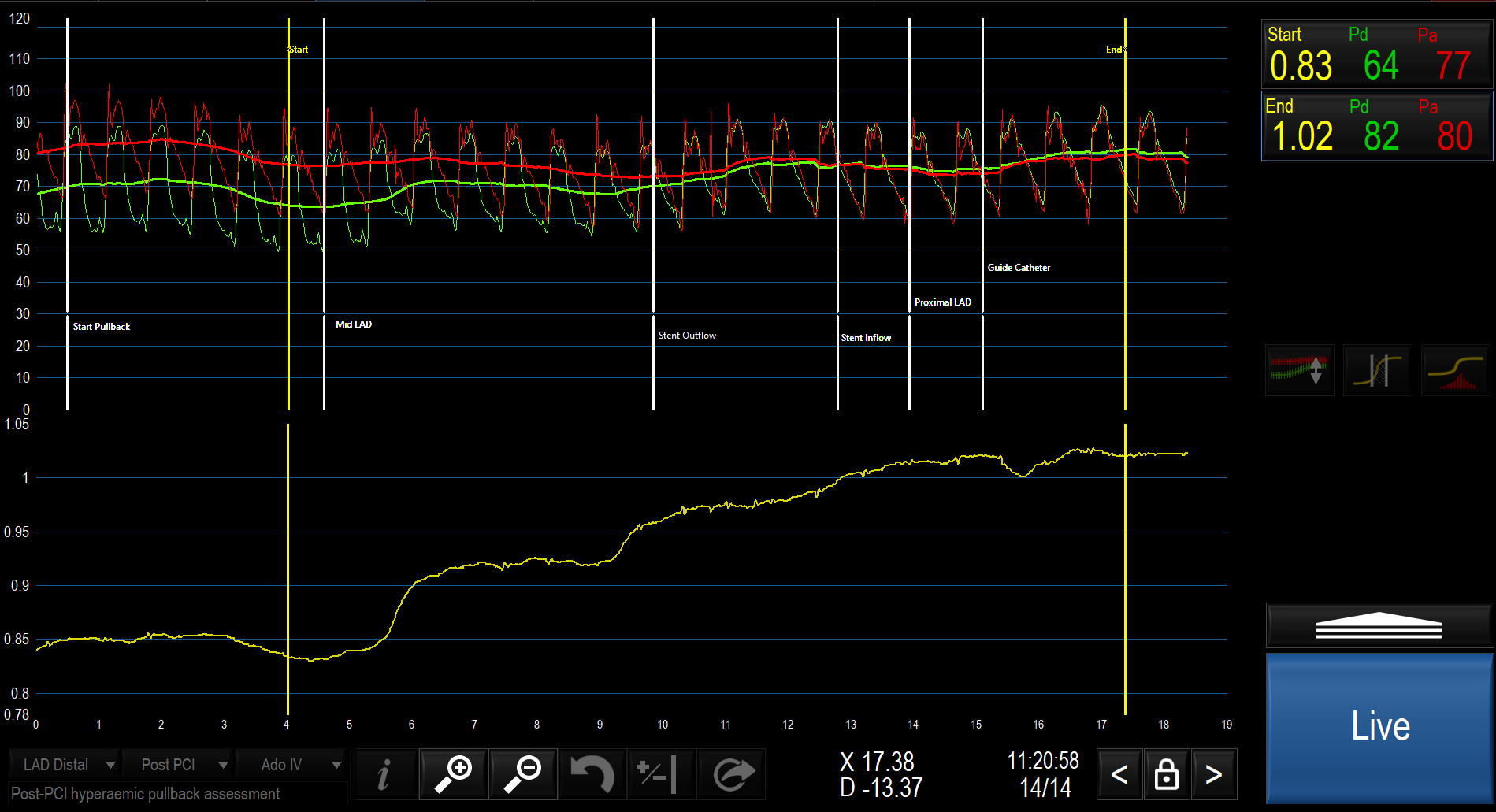 |
| --- |
| **Case 7.** Post-PCI FFR 0.81. 3.5/18mm stent proximally post-dilated with 3.75mm NC balloon to 16atm. HTG of 0.04 units. Focal pressure drop at stent outflow with larger focal drop of 0.09 units distally. Operator unwilling to stent either the disease at stent outflow or the more distal focal lesion as FFR already >0.80 and distal lesion was located at a bifurcation point. |

| 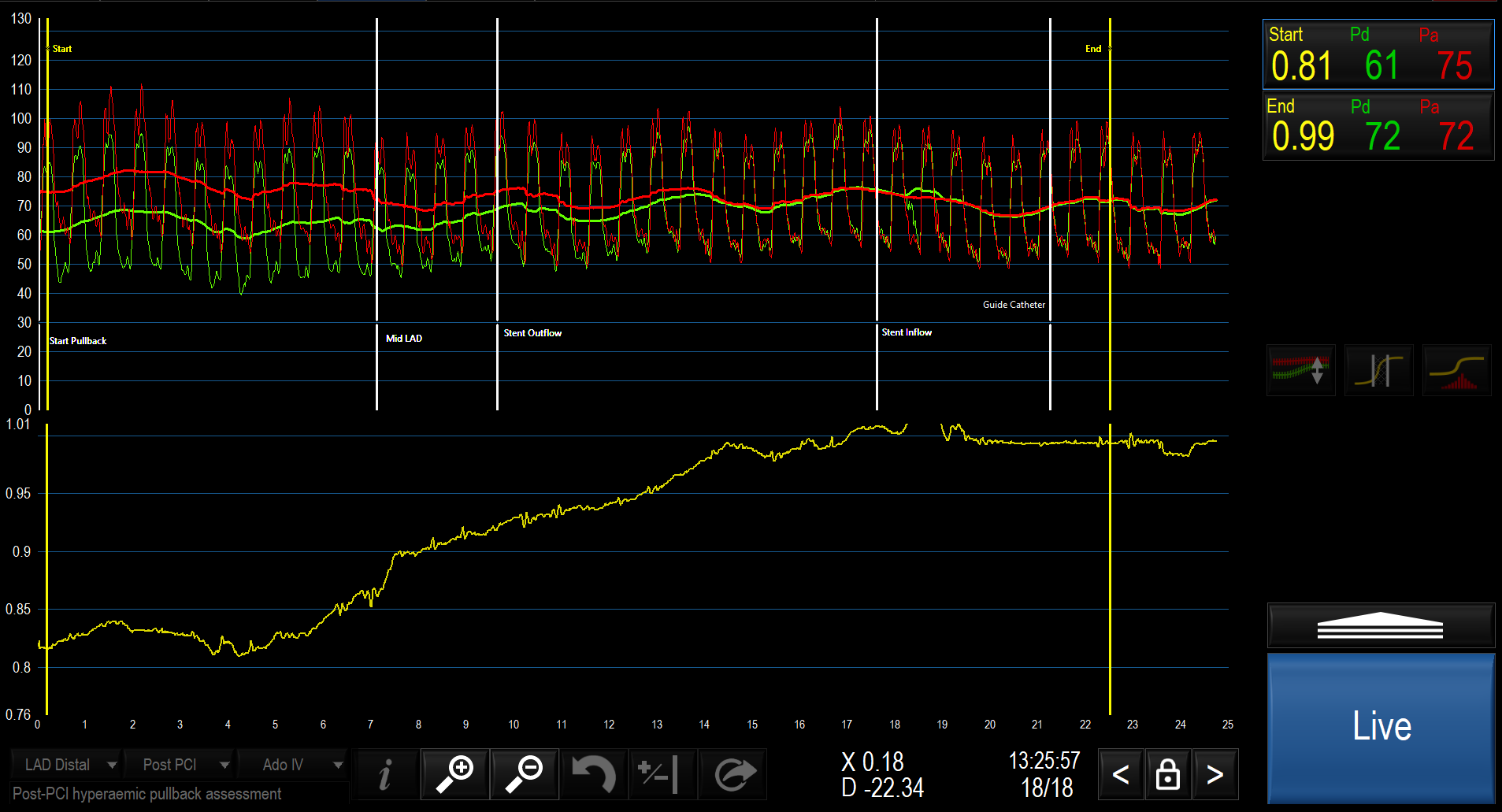 |
| --- |
| **Case 8.** Post-PCI FFR 0.81. 3.0/48mm stent post-dilated with 3.5mm NC balloon to 14atm. Diffuse residual gradient with HTG of 0.07 units. Operator felt stent had been adequately post-dilated, reluctant to post-dilate with larger balloon and felt diffuse HTG was attributed to stent length. |

| 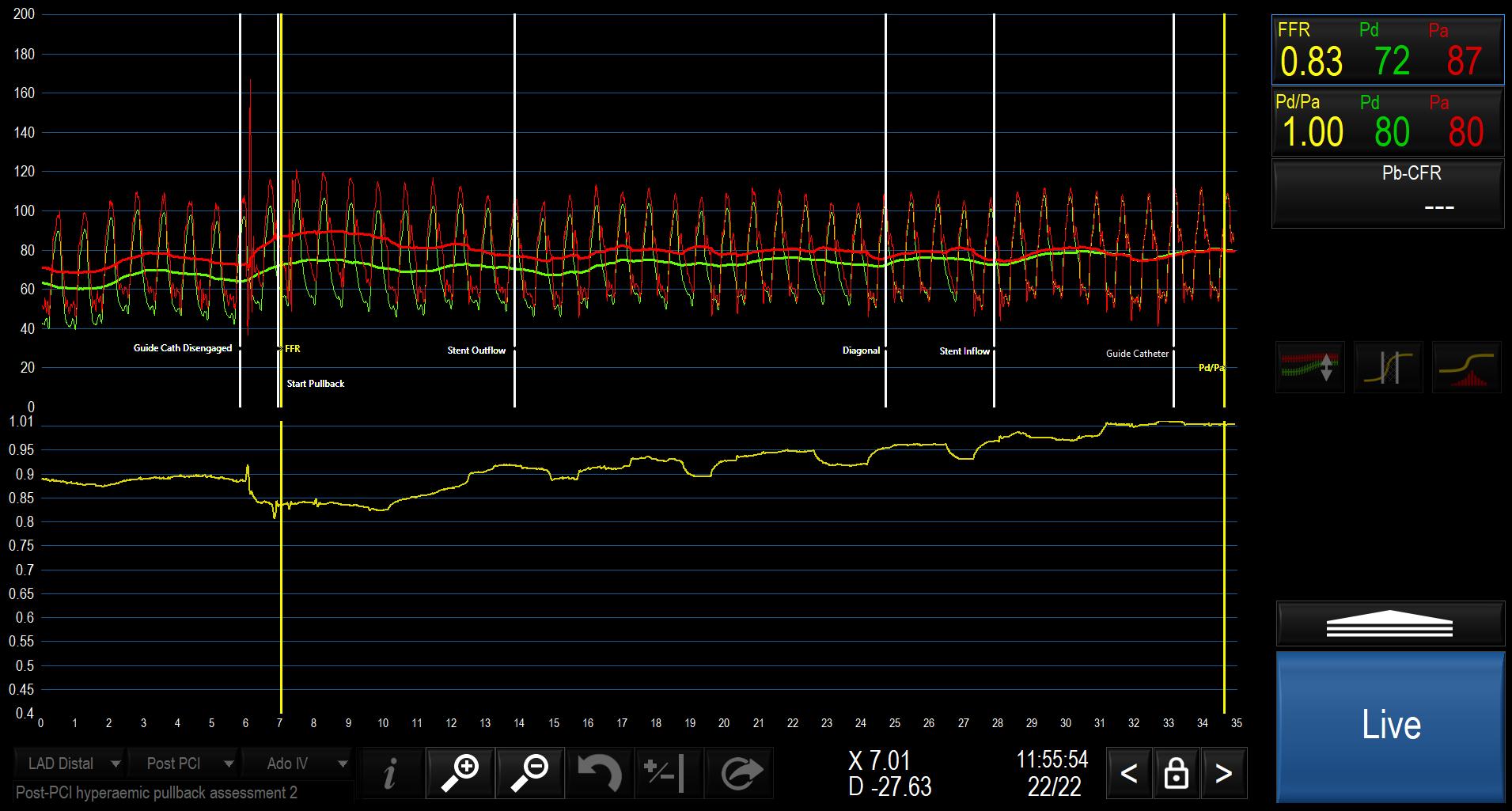 |
| --- |
| **Case 9.** Post-PCI FFR 0.82. 3.5/23mm stent deployed at target lesion. Overlapping 3.0/48mm stent distally to cover long segment of diffuse disease. Post-dilation of both stents with 3.5mm NC balloon to 16atm. Further post-dilation with 3.75mm NC balloon to 20atm in proximal segment for optimisation of stent above the origin of main diagonal branch. Diffuse residual gradient with HTG of 0.05 units. Operator felt stents had been adequately post-dilated and unlikely to be able to further reduce HTG due to length of stented segment. |

| 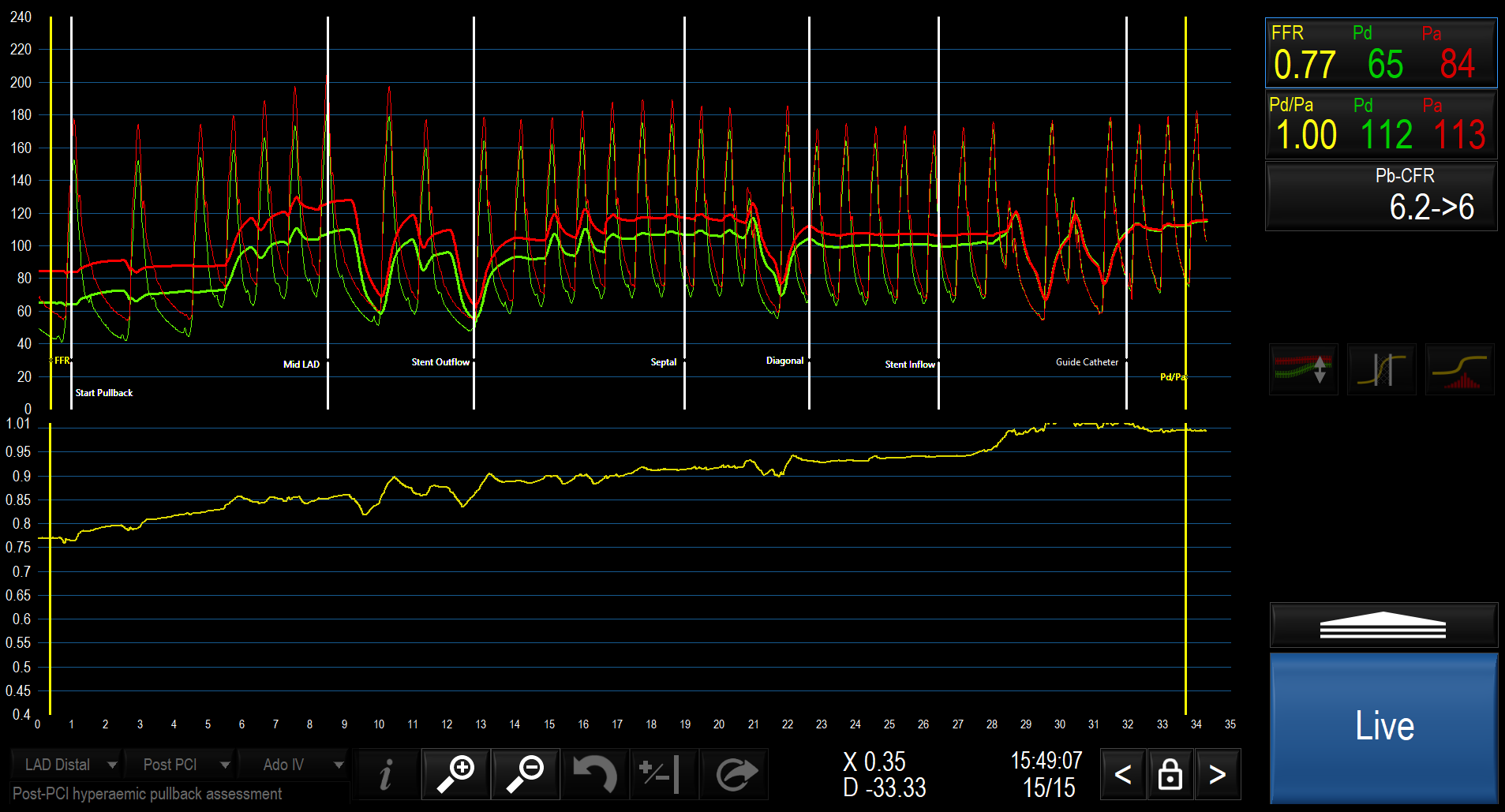 |
| --- |
| **Case 10.** Post-PCI FFR 0.78. 3.0/38mm stent post-dilated with 3.25mm NC balloon to 18atm. Diffuse residual gradient with HTG of 0.05 units. Operator felt stent had already been appropriately post-dilated and that further intervention would not reduce HTG significantly. Note pressure waveform artefact due to intermittent heart block. |

| No post-PCI pullback performed |
| --- |
| **Case 11.** Post-PCI FFR 0.71. Medina 1,1,0 bifurcation lesion involving proximal LAD and diagonal branch. Culotte technique. 2.75/38 stent from LAD into diagonal. Proximal optimisation with 3.5mm NC balloon. 3.5/38mm stent in LAD. Final kissing balloon angioplasty with 3.5/ NC balloon in LAD (10atm) and 2.75mm NC balloon into diagonal (10atm). Computer software failure mandating system restart after during post-PCI measurements. Operator unwilling to remove pressure wire to re-zero or continue with repeat measurements and optimisation protocol. No post-PCI pullback performed and therefore no optimisation attempted. |

| 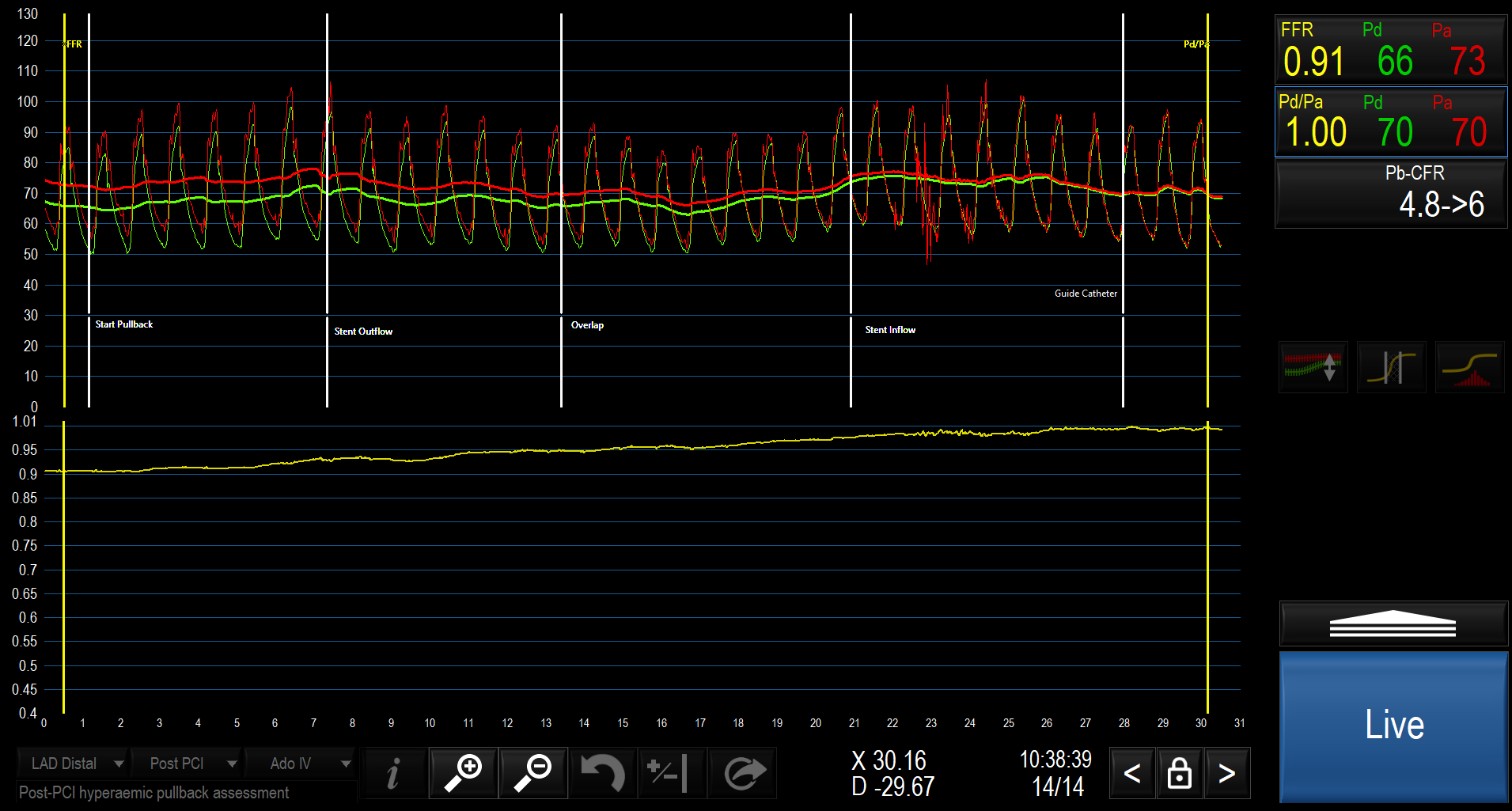 |
| --- |
| **Case 12.** Post-PCI FFR 0.87. 3.5/20mm stent at target lesion. Additional 3.0/32mm stent overlapping distally to treat second lesion downstream. Distal-to-mid stent segment post-dilated with 3.25 NC balloon (18-22atm) and proximal segment with 3.75/15mm NC balloon (18-20atm) Diffuse residual gradient with HTG of 0.05 units. Operator felt stent had been adequately post-dilated and that further dilatation with larger balloons may be harmful |

| 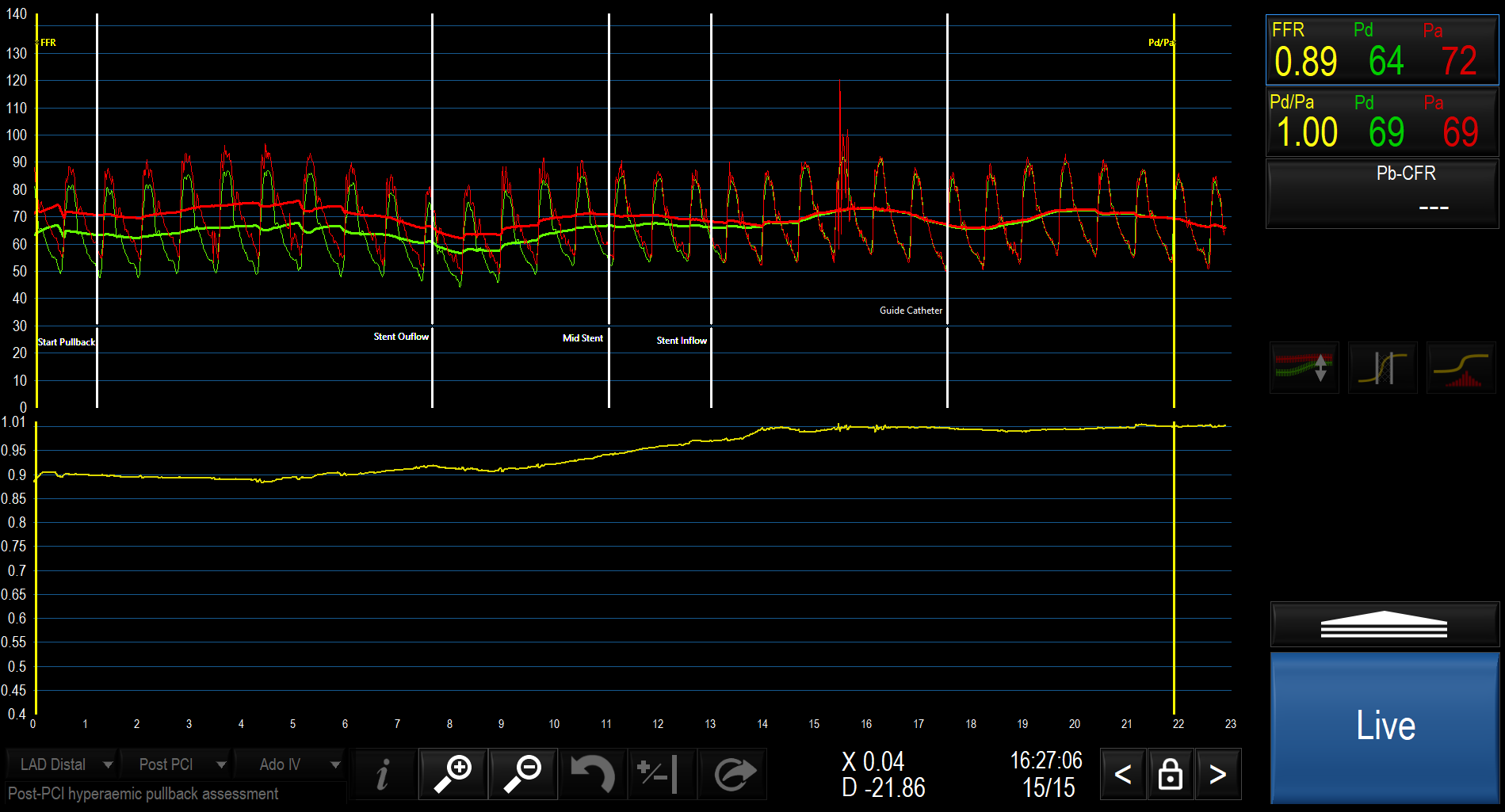 |
| --- |
| **Case 13.** Post-PCI FFR 0.84. 3.5/48mm stent post-dilated with a 3.5mm NC balloon to 18atm. Diffuse residual gradient with HTG of 0.06 units. Operator believed stent was adequately post-dilated and that further expansion attempts with a larger balloon would not significantly reduce the gradient in a long stent and may be hazardous. |

| 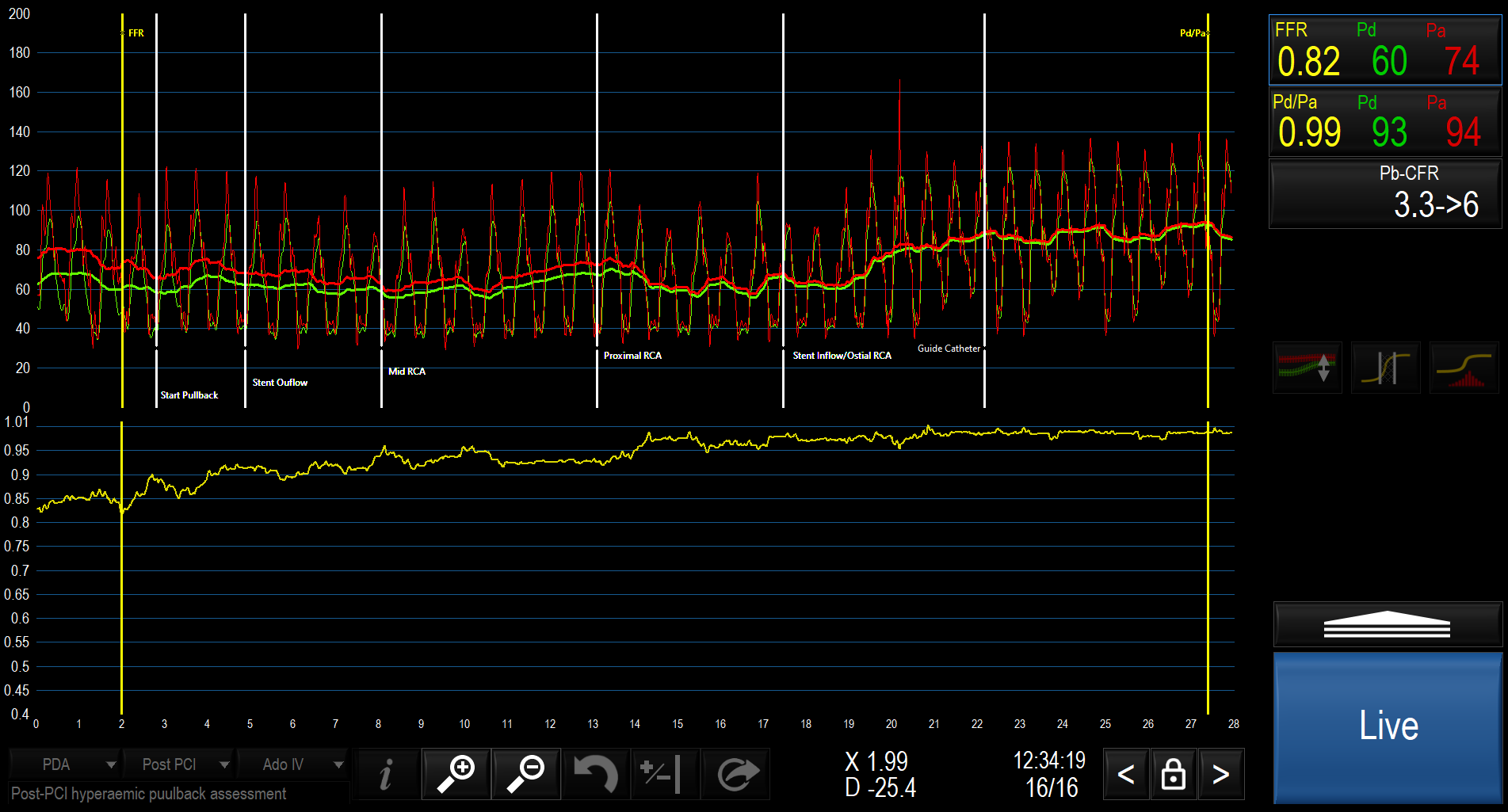 |
| --- |
| **Case 14.** Post-PCI FFR 0.80. 3.0/38mm stent deployed at target lesion. Additional 3.0/23mm stent overlapping proximally to cover disease at stent inflow. Post-dilation with 3.25mm NC balloon up to 20atm. Diffuse residual gradient with HTG of 0.06 units. Operator felt stent had been adequately post-dilated and that residual HTG of was related to total stent length of 60mm and unlikely to change with further post-dilation. |

| 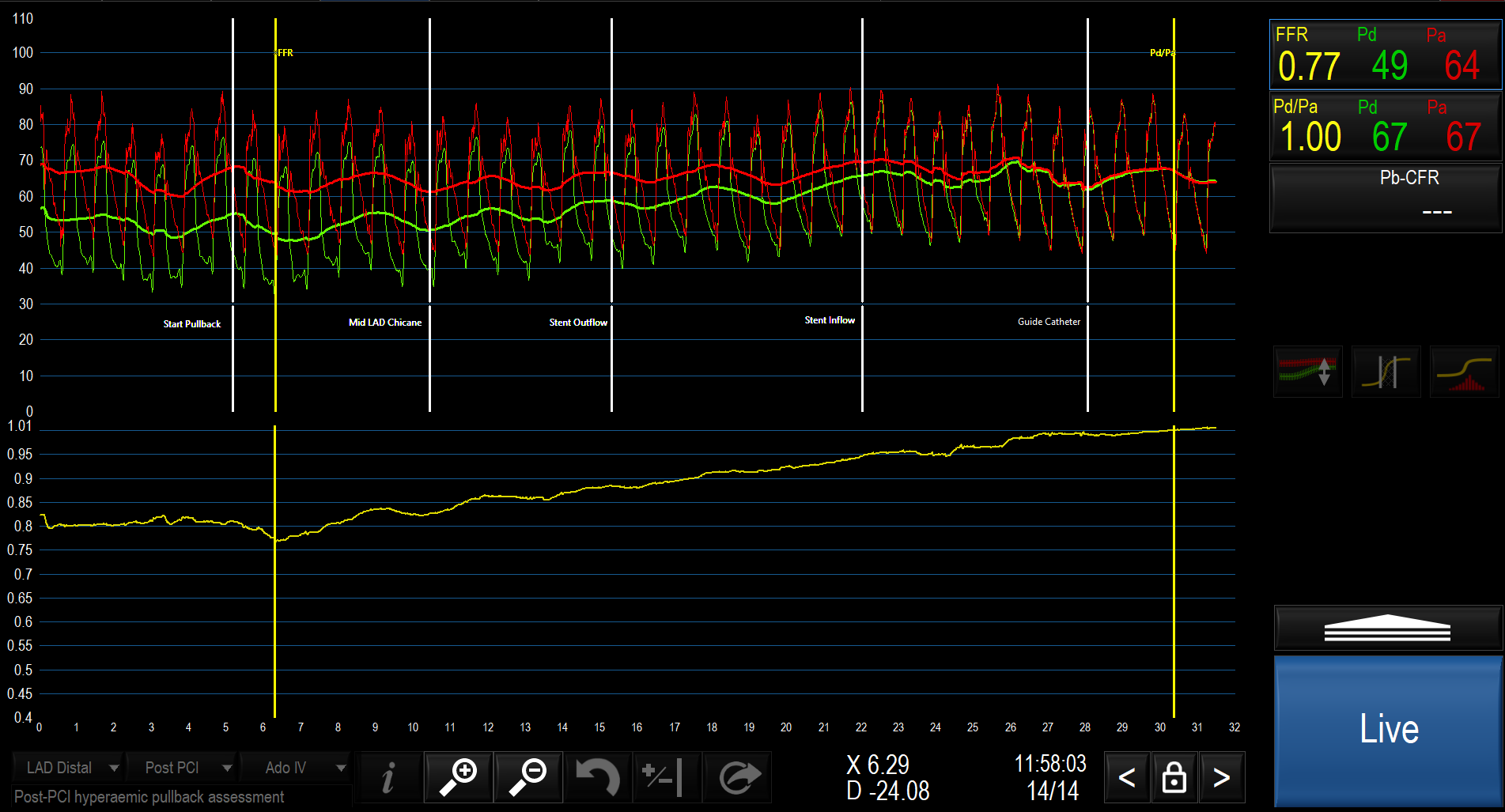 |
| --- |
| **Case 15.** Post-PCI FFR 0.76. 3.5/33mm stent. Post-dilated with 3.75/12mm NC balloon (16atm) distally and 4.0/20mm NC balloon (18atm) proximally. Diffuse residual gradient with HTG of 0.07 units. Operator felt stent had been adequately post-dilated and that further aggressive efforts at expansion may be harmful. |

| 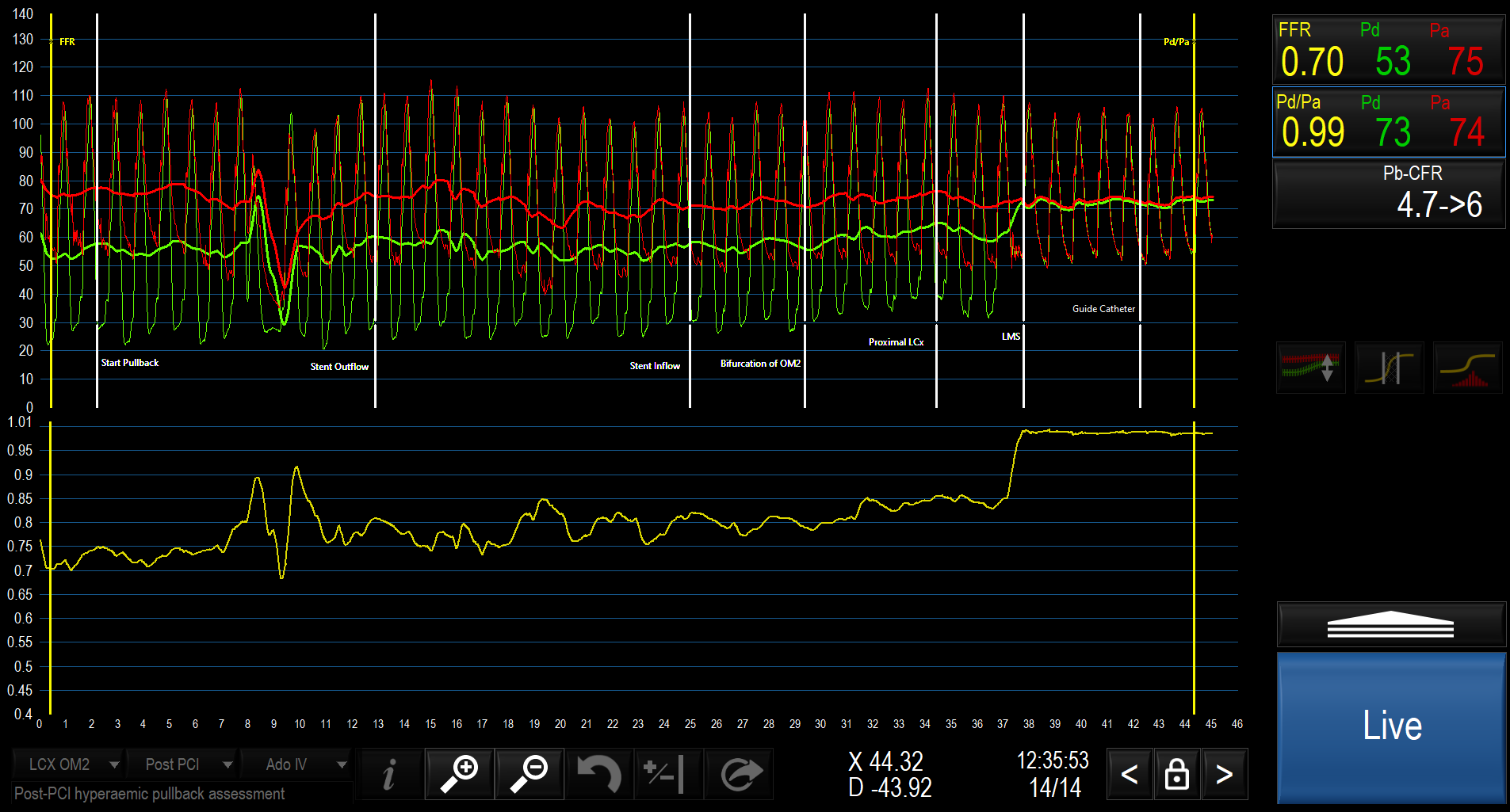 |
| --- |
| **Case 16.** Post-PCI FFR 0.70. 3.0/28mm stent post-dilated with 3.25mm NC balloon to 20atm. Diffuse residual gradient distally with HTG of 0.02 units. Focal step-up of 0.16 units proximally at ostial vessel back in to left main stem. Operator did not wish to proceed to PCI of left main stem. Pressure waveform artefact distally relating to ectopic heartbeat. |

| 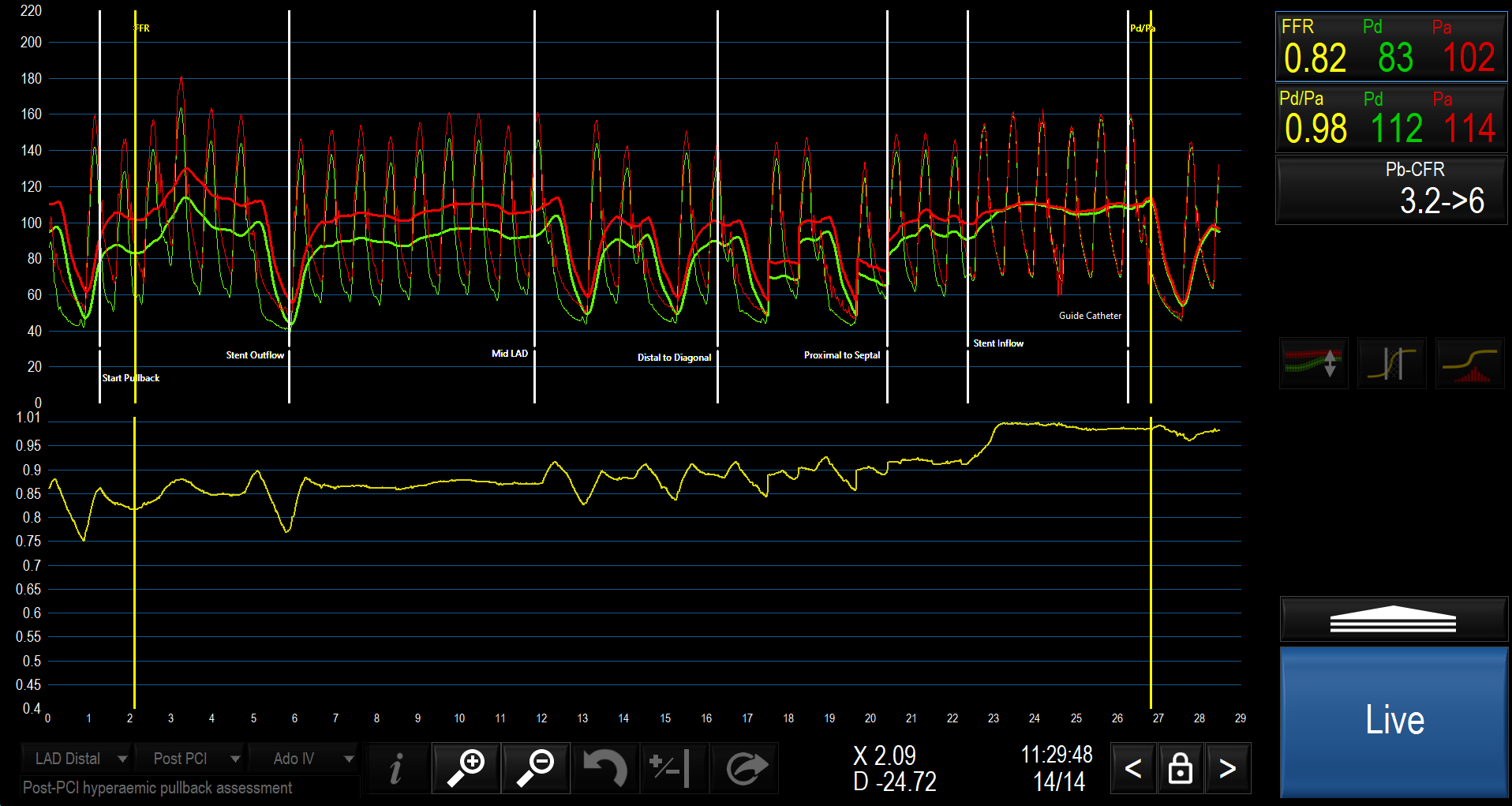 |
| --- |
| **Case 17.** Post-PCI FFR 0.82. 3.5/48mm stent deployed at target lesion. Additional 3.5/15mm stent overlapped proximally to cover inflow disease. Post-dilated with 3.75mm NC balloon up to 14atm. Diffuse residual gradient distally with HTG of 0.05 units. Relatively focal step-up of 0.07 units back into left main stem. Operator believed stent was adequately post-dilated and borderline residual HTG related to stent length. Did not feel stenting back into LMS was warranted. Note pressure waveform artefacts related to ectopic heart beats. |

| 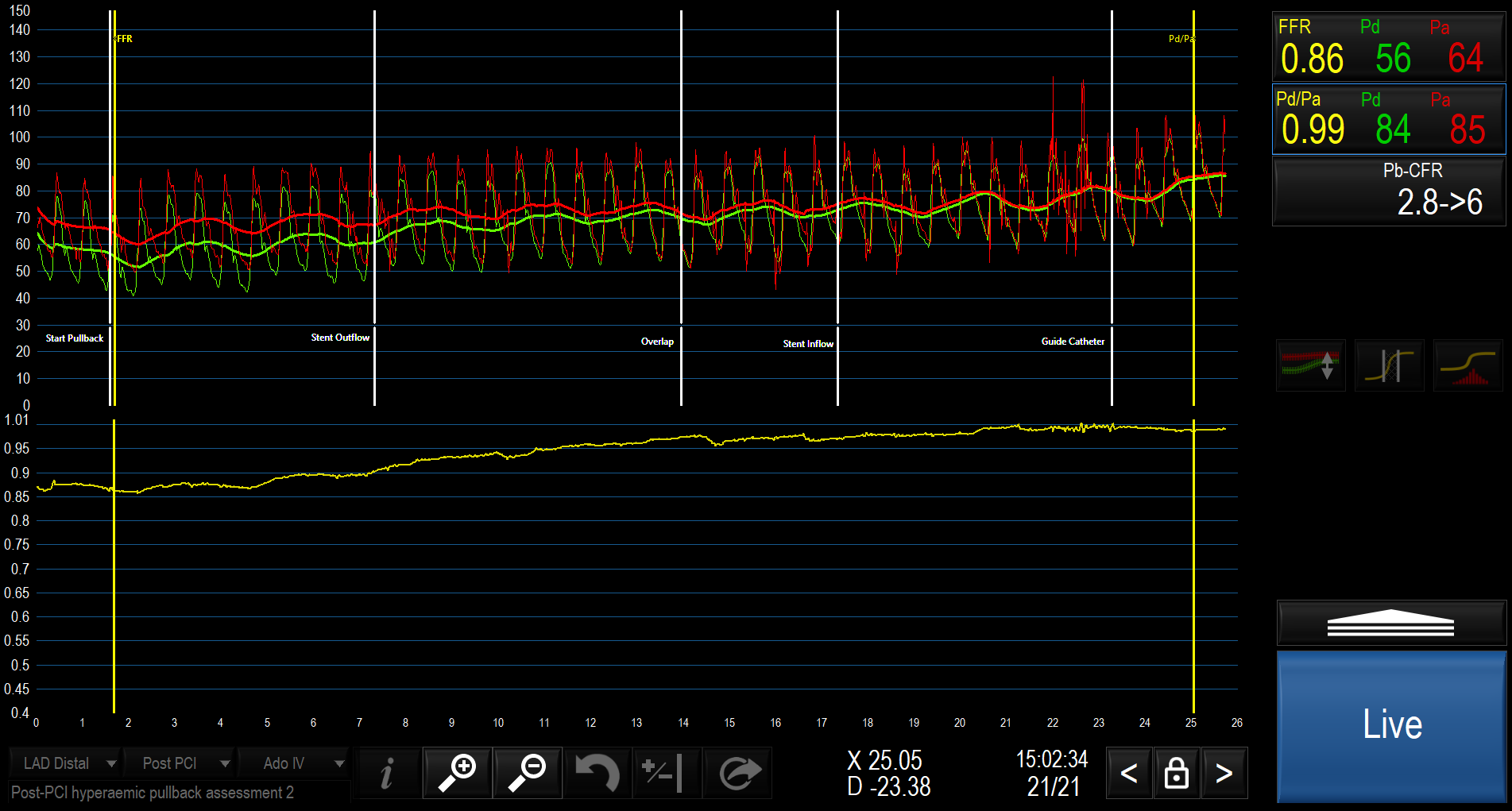 |
| --- |
| **Case 18.** Post-PCI FFR 0.80. 3.0/38mm stent to target lesion. Additional 4.0/20mm stent overlapping proximally to cover disease at stent inflow. Sequential post-dilation: 3.5/15mm NC balloon (16-20atm) to first stent then 4.0/12mm NC balloon (18-24tm) to second stent and overlap. Finally, a 3.25 NC balloon (14-16atm) applied to an area of eccentric under-expansion at stent overlap. Diffuse residual gradient with HTG of 0.07 units. Following high-pressure post-dilations guided by Intravascular Ultrasound (IVUS) imaging, the operator was satisfied with stent deployment and felt no further optimisation achievable by additional post-dilation. Residual gradient felt to be due to length of stented segment. |

| 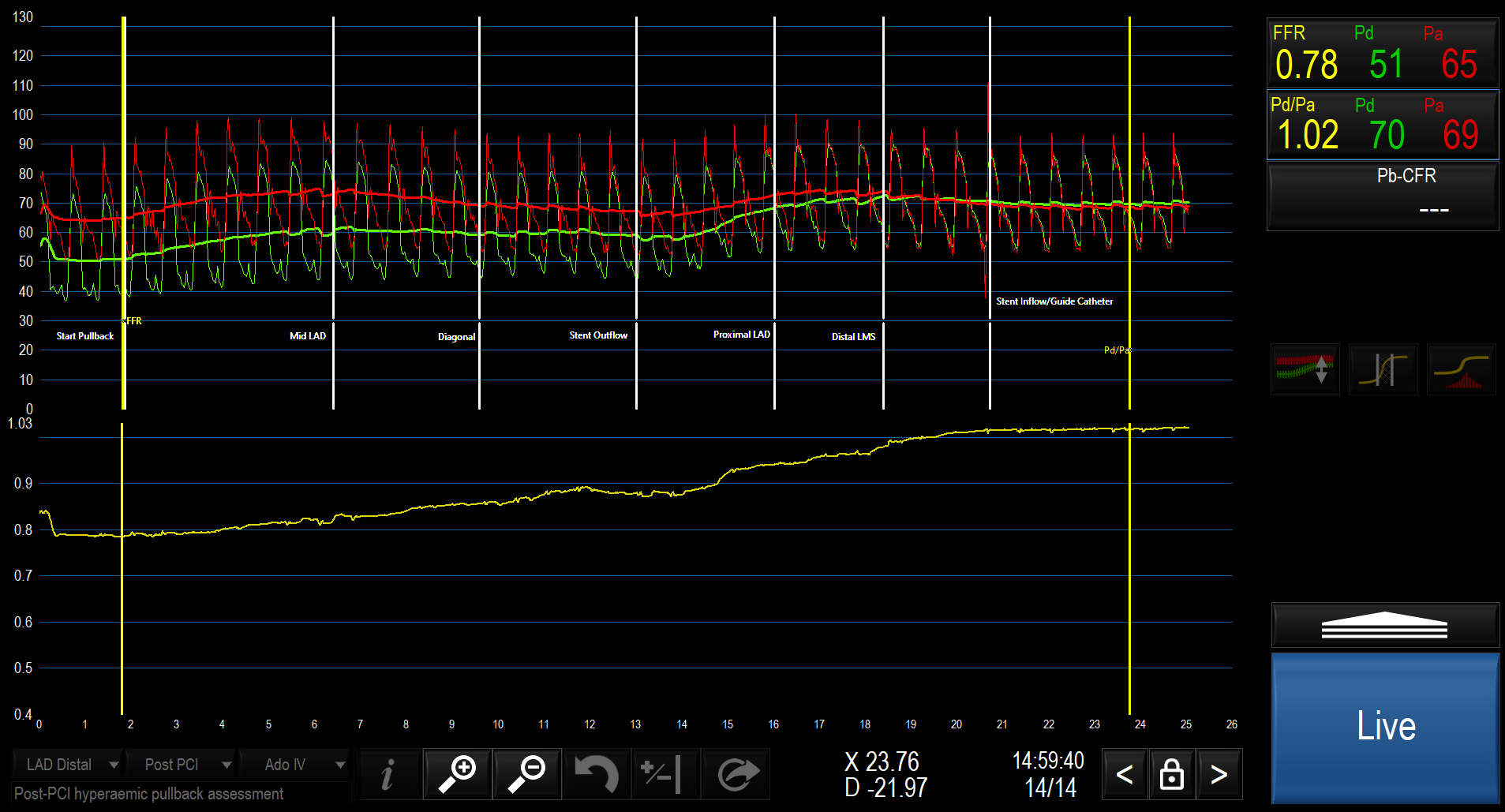 |
| --- |
| **Case 19.** Post-PCI FFR 0.77. 3.5/38mm stent deployed from LAD back into Left Main Stem. Sequential post-dilation initially with 3.5/15mm NC balloon (16atm) in LAD and 4.5/8mm NC balloon (10-14atm) in LMS. Following IVUS assessment, a 4.0/12mm NC balloon applied to segment in proximal LAD (16atm) and a 5.0/12mm NC balloon (8-12atm) to segment in left main. Diffuse residual gradient with HTG of 0.07 units. Based on IVUS appearances, operator was satisfied stent had been appropriately post-dilated with large diameter NC balloons to high pressure and further aggressive post-dilation may have been harmful |

| 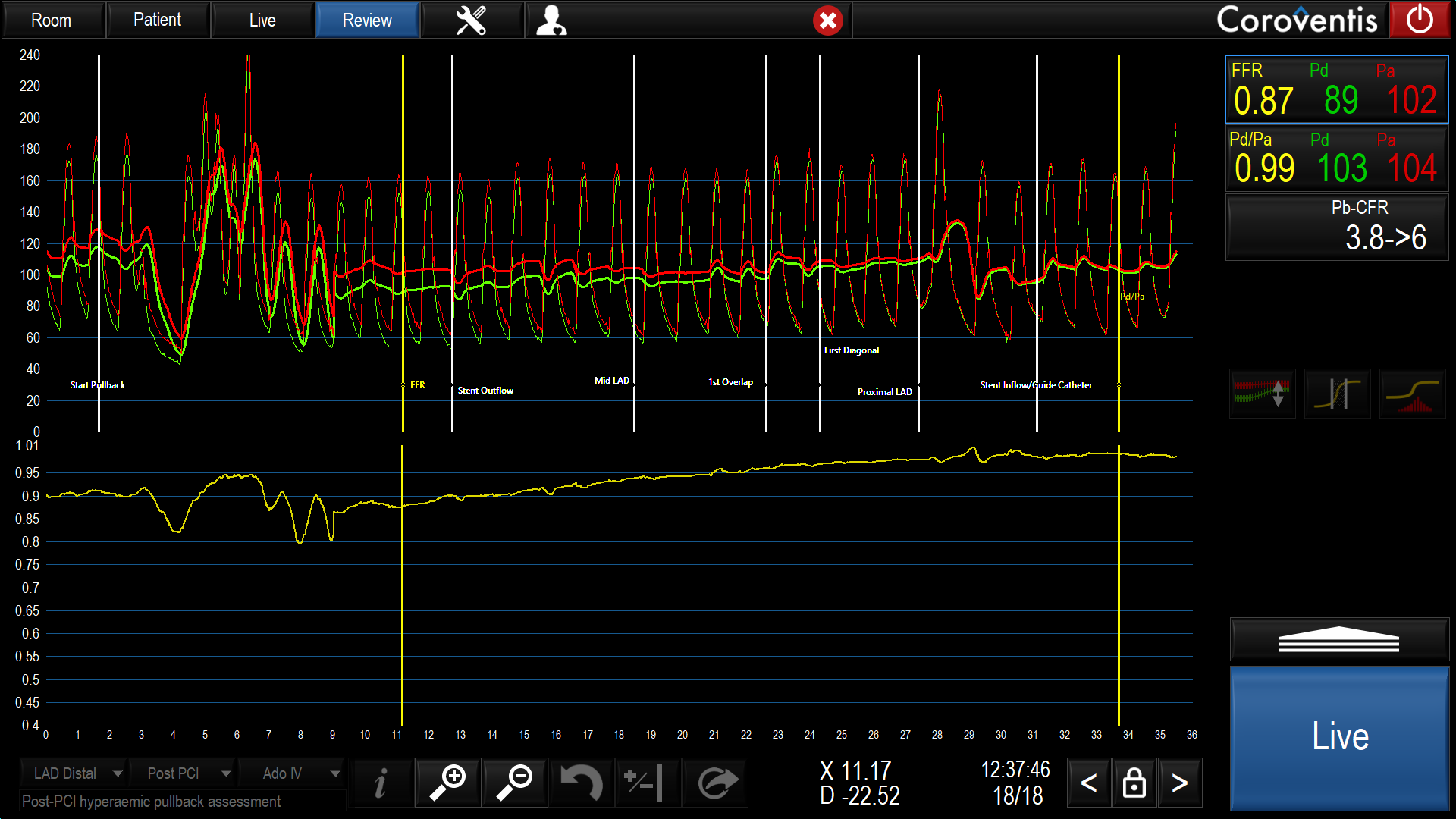 |
| --- |
| **Case 20.** Post-PCI FFR 0.87. 3.0/38mm stent deployed at target lesion in LAD back into left main stem. Additional 2.75/48mm stent deployed to overlap distally due to distal stent edge dissection and to treat diffuse outflow disease with further discrete lesion downstream. Sequential post-dilation of stents with 3.0/15mm NC balloon (14-18atm), followed by 3.5/20mm (12-16atm) and 4.0/15mm NC (14atm) in proximal LAD segment. Finally, following OCT assessment, a 4.0/12mm NC balloon was applied to LMS segment at high pressure (16-18atm). Diffuse residual gradient with HTG of 0.08 units. Patient intolerant of further adenosine, satisfactory, no further intervention attempted and result accepted. Note pressure waveform artefacts related to ectopic beats. |
